# Selected traditional Chinese medicines for treating amyotrophic lateral sclerosis: a Bayesian network meta-analysis based on randomised controlled trials

**DOI:** 10.3389/fphar.2026.1706211

**Published:** 2026-10-08

**Authors:** Chen Shen, Xiao-Ti Wu, Heng Yin, Jing-Ling Zuo, Chen-Hao Zhang, Feng Cao, Ying Zhang, Zi-Yu Tian, Nicola Robinson, Jian-Ping Liu

**Affiliations:** 1 Centre for Evidence-Based Chinese Medicine, Beijing University of Chinese Medicine, Beijing, China; 2 Institute of Acupuncture and Moxibustion, China Academy of Chinese Medical Sciences, Beijing, China; 3 Institute of Health and Social Care, London South Bank University, London, United Kingdom; 4 Centre for Evidence Based Medicine, Hospital of Chengdu University of Traditional Chinese Medicine, Chengdu, China

**Keywords:** amyotrophic lateral sclerosis, Bayesian network meta-analysis, Chinese herbal medicine, indirect comparison, rare disease, traditional Chinese medicine

## Abstract

**Objective:**

To assess the effectiveness of specific Traditional Chinese Medicine (TCM) therapies for Amyotrophic Lateral Sclerosis (ALS) using Bayesian network meta-analysis.

**Methods:**

PubMed, the Cochrane Library, EMBASE, Web of Science, and four Chinese databases up to 15th December 2024 was searched, including RCTs on adult Amyotrophic Lateral Sclerosis patients treated with TCM therapies (Chinese herbal medicine, preparations, non-pharmacological treatments) alone or combined with conventional treatments. Controls included no intervention, placebo, conventional treatments, or combinations. Primary outcomes were functional status (ALSFRS, ALSFRS-R, AALSS, m-Norris scales). Bayesian network meta-analysis compared treatment effects and calculated SUCRA values.

**Results:**

Thirty-five RCTs involving 27 TCM therapies were included. No significant differences in functional status or quality of life were found among TCM therapies, ICT, Riluzole alone, and Riluzole plus placebo. For functional status, Shenmai injection + ICT (SUCRA 0.735 for ALSFRS, 0.870 for ALSFRS-R), Jiweiling injection + placebo (SUCRA 0.868 for ALSFRS), Jiawei Sijunzi decoction alone (SUCRA 0.739 for ALSFRS-R), Acupuncture + rehabilitation exercises + Riluzole (SUCRA 0.928 for AALSS, 0.863 for m-Norris) and Shenzhe Jiangqi powder + placebo (SUCRA 0.860 for m-Norris) had the highest probabilities of being superior treatment. For quality of life, Thunder fire moxibustion + Riluzole and Jiweiling injection + placebo for ALSAQ-40 were favorable.

**Conclusion:**

Despite no significant differences in functional status among TCM therapies, ICT, Riluzole alone, and Riluzole plus placebo, Jiweiling injection, Shenmai injection, Jiawei Sijunzi decoction, and Shenzhe Jiangqi powder may be regarded as candidate preparations for ALS patients. Future research should focus on real-world data to explore long-term effects and practical benefits.

**Systematic Review Registration:**

https://inplasy.com/inplasy-2024-8-0050/, identifier INPLASY202480050.

## 1 Introduction

Amyotrophic Lateral Sclerosis (ALS) is a neurodegenerative disease that primarily affects upper and lower motor neurons, leading to progressive muscle weakness, atrophy, and paralysis (Feldman et al., 2022). The etiology of ALS is complex and not fully understood, but it is generally believed to involve the interplay of genetic and environmental factors (Ryan et al., 2019). The diagnosis of ALS is primarily based on clinical presentation and electrophysiological examinations (Brooks et al., 2000; de Carvalho et al., 2008) with the revised El Escorial criteria, Awaji criteria, and Gold Coast criteria serving as the principal diagnostic standards. These criteria necessitate the presence of clinical signs indicating damage to upper and lower motor neurons across two or more neural segments, which progressively worsen over time, while ruling out other diseases that could mimic these symptoms (Brooks et al., 2000; Shefner et al., 2020; Zuo et al., 2021). The pathogenesis of ALS involve multiple complex biological processes, such as protein misfolding and aggregation, mitochondrial dysfunction, oxidative stress, and neuroinflammation. Mutations in genes like SOD1, TDP-43, and FUS have been identified in some patients (Ross and Poirier, 2004; Peggion et al., 2022; Carata et al., 2023; Zuo et al., 2021; Ilieva et al., 2023), highlighting the significance of genetic factors. Globally, ALS has a prevalence of approximately 4.1–8.4 cases per 100,000 individuals, with an annual incidence ranging from 0.6 to 3.8 cases per 100,000 individuals. It is more common in males and typically manifests in middle-aged and older adults. Variations across countries and regions may arise from differences in genetic backgrounds, environmental factors, and diagnostic criteria (Wolfson et al., 2023; Longinetti and Fang, 2019).

ALS is a fatal disease with an average survival time of 2–5 years, although a small proportion of patients may survive for more than 10 years (Mehta et al., 2017; Testa et al., 2004; Bond et al., 2020). Patients experience a gradual loss of motor function, leading to respiratory failure (Goutman et al., 2022) and significant impacts on quality of life, including limb weakness and impairments in speech, swallowing, and respiratory function. These challenges impose substantial physical, psychological, and economic burdens on both patients and their families (James et al., 2022; Achtert and Kerkemeyer, 2021; Yu et al., 2024). Currently, there are four FDA-approved drugs for ALS in the United States: Riluzole (U.S. Food and Drug Administration, 2022a), Edaravone (U.S. Food and Drug Administration, 2022a), RELYVRIO (U.S. Food and Drug Administration, 2022b), and Tofersen (U.S. Food and Drug Administration, 2023). Outcome measures and evaluation methods for ALS include muscle strength measurement, respiratory function assessment, survival time, and disability monitoring (Paganoni et al., 2014). Among these, the ALS Functional Rating Scale-Revised (ALSFRS-R) is the most frequently utilized functional assessment tool for evaluating motor abilities and daily living skills (Feldman et al., 2022; Cedarbaum et al., 1999), alongside additional evaluation indicators such as biomarkers, ROADS, etc (Hartmaier et al., 2022).

According to a cross-sectional survey in China, more than 99% of ALS patients use integrative therapies (Pan et al., 2013a). The most frequently employed integrative therapies in China include vitamins, Chinese herbal decoctions, Chinese herbal compounds, massage therapy, and acupuncture, with more than 90% of patients relying on herbal medicines. ALS is predominantly classified as a “atrophy-flaccidity disease” in traditional Chinese medicine (TCM) (Liao et al., 2022). The characteristics of atrophy-flaccidity, including muscle weakness, muscle atrophy, and impaired limb mobility, align with the primary symptoms of ALS. ALS is characterized by the location of disease in the spleen and kidney, and the nature of disease includes qi deficiency, wind, and phlegm-dampness (Teng et al., 2023). In animal and *in vitro* experiments in ALS, some Chinese medicine therapy has achieved positive results. Buzhong Yiqi decoction improves the function of the spinal cord and muscles among mice (Cai et al., 2019) and Jiweiling injection enhances motor neuron activity (Wang et al., 2007). Acupuncture is associated with increased motor neuron survival, delayed loss of motor performance (Yang et al., 2010), and decreased inflammatory markers (Yang et al., 2010; Jiang et al., 2011). A qualitative study found that although ALS patients and their families have not discovered a cure using alternative medicine, they actively use it to manage various symptoms and remain open to new possibilities (Kim et al., 2016). The use of some herbal and other botanical medicines have been reported to benefit patients in terms of overall survival at 18 months and delayed activity dysfunction (Song et al., 2022a).

Clinical studies have demonstrated that short-term adjunctive use of Chinese herbal medicine (CHM) such as Jiweiling injection, Shenmai injection, and Jiawei Sijunzi decoction, improves ALS functional rating scales (ALSFRS) scores and demonstrates beneficial clinical effects compared with placebo or Riluzole alone (Liao et al., 2022; Pan et al., 2013b; Ma, 2006; Zhang, 2020). These effects include delaying the progression of activity dysfunction (Wang, 2007; Cai, 2011; Zhang, 2020; Luo et al., 2018) and mitigating the decline in respiratory muscle strength (Wang, 2007; Wang et al., 2023). Acupuncture has the potential to improve ALS symptoms such as fatigue and neurological deficits and reduce relapse (Wang et al., 2023).

Therefore, this systematic review and network meta-analysis aimed to assess and compare different TCM therapies used alone or in combination with conventional medicine in managing ALS. On the basis of the findings, it was hoped to provide evidence to guide clinical decision-making and optimize TCM strategies for patients with ALS.

## 2 Methods

The systematic review and network meta-analysis followed the Preferred Reporting Items for Systematic Reviews and Meta- Analyses for Network Meta-Analysis recommendations (Hutton et al., 2015). The protocol was registered with the International Platform of Registered Systematic Review and Meta-analysis Protocols (INPLASY) under the number INPLASY202480050 (https://inplasy.com/inplasy-2024-8-0050/).

### 2.1 Eligibility

Adults with age over 18 years who were diagnosed with ALS were included. Diagnostic criteria based on any version of the consensus criteria, such as the El Escorial criteria (2000), Awaji algorithm (2008), or Gold Coast criteria (2020), was considered acceptable. Adapted diagnostic criteria based on these standards, commonly employed in various countries, was also permissible. There was no restriction on the race, or gender of the participants. TCM therapies were employed either independently or as adjunctive therapy alongside conventional medicine. These TCM interventions encompass Chinese herbal medicine or preparations, as well as non-pharmacological approaches like acupuncture or massage. There were no specific restrictions regarding the dosage or formulation of herbal medicines. Eligible control therapy included one or a combination of a placebo, no treatment, TCM therapies and conventional medicine.

Primary outcomes included functional status assessed using the ALS functional rating scales (ALSFRS) or the revised ALS functional rating scales (ALSFRS-R), Appel ALS Score (AALSS), or the modified Norris scale (m-Norris). Secondary outcomes included: 1) Survival time, including tracheostomy-free survival or overall survival. Tracheostomy-free survival is defined as time to death, tracheostomy, or permanent non-invasive positive pressure ventilation. 2) Quality of life: Assessed using specialized scales such as the ALS Specific Quality of Life Instrument (ALSSQOL), ALS Assessment Questionnaire-40 (ALSAQ-40) or general scales such as the MOS Item Short-form Health Survey (SF-36) or the Barthel index. 3) Respiratory Function: Measured using Vital Capacity (VC) or Forced Vital Capacity (FVC). 4) Symptom improvement or relief measured by validated scales or instruments. 5) Motor neuron loss: Assessed via motor unit number estimation (MUNE) or other neurophysiological tests. 6) Cognitive and behavioral impairment: Measured using tools such as the ALS Cognitive Behavioral Screen (ALS-CBS). The safety outcome included adverse events.

### 2.2 Information source

The following electronic databases were searched from their inception to 15th December 2024, using a predefined search strategy (Supplement 1): PubMed, the Cochrane Library, EMBASE, Web of Science, China National Knowledge Infrastructure (CNKI), Wanfang database, Chinese Scientific Journal Database (VIP) and Chinese biomedical literature database (CBM). The reference lists of all full-text papers were hand searched for additional relevant reports. Searches were limited to randomized controlled trials (RCTs) in all languages. The six authors in pairs (C Shen, XT Wu, CH Zhang, H Yin, F Cao, JL Zuo) screened the studies and provided data from previously published studies. Data included study ID, study design, age, sample size, course of disease, diagnostic criteria, comparisons, outcomes and outcome measures. In addition to general trial characteristics, we extracted information required for reproducibility of the TCM interventions, including the name of the preparation, dosage form, route and duration, composition using pharmacopoeial Latin names with pinyin in parentheses where identifiable, acupoint names with standard international codes where reported, producer or preparation source, batch or approval number, quality-control or pharmacopoeial standard, and preparation/processing method. When these details were absent from the original report or could not be reliably recovered from the available full text, they were recorded as not reported.

### 2.3 Quality assessment

The Cochrane Risk of Bias 2.0 (RoB 2.0) was employed to evaluate the methodological quality of included RCTs. The six authors in pairs independently assessed the quality and consensus were reached by discussion with a third party (Liu JP) in case of discrepancy. Five areas were assessed in RoB 2.0: bias generated in the random process, bias deviating from the established intervention, bias of missing outcome data, bias of outcome measurement and bias of selective reporting of results.

### 2.4 Statistical analysis

Using direct or indirect comparisons and cumulative ranking probability, network meta-analysis can be evaluated for relative advantages and disadvantages. This study was based on the Bayesian network meta-analysis and used R 4.4.0. A random-effects consistency model was applied. Four Markov chains were run, with 20,000 iterations for adaptation and 50,000 iterations for sampling. Default prior distributions implemented in the gemtc package were used. A risk ratio (RR) with a 95% confidence interval (CI) were calculated for dichotomous variables. In the case of continuous variables, we calculated the mean difference (MD) or standard mean difference (SMD) the with 95% CI. The I^2^ test and χ^2^ test was used to assess heterogeneity. All effect sizes were analyzed using a random effect model because it gives similar weights to studies with different sample sizes and substantial heterogeneity would be expected between studies.

Network plots were used to illustrate the geometry of the evidence network, where nodes represent different interventions and edges represent direct comparisons between interventions. The size of nodes is proportional to the sample size, and the thickness of edges corresponds to the number of studies. In cases where the diagram contained a closed loop, the inconsistency test was used to evaluate the consistency between the results of direct and indirect comparisons. In the absence of a closed loop, the consistency model was directly used for analysis. The transitivity assumption was assessed by comparing the distribution of potential effect modifiers across treatment comparisons within each network. Potential effect modifiers included age, disease duration, co-interventions and outcome measures. Each outcome was ranked using the surface under the cumulative ranking (SUCRA) curve, with a greater SUCRA value indicating a better effect.

## 3 Results

### 3.1 Search results and characteristics of included trials

A total of 1,650 references were initially identified (Figure 1). Thirty-five RCTs were ultimately included (Cai et al., 2023; Cai, 2011; Chen et al., 2004; Chen and Yang, 2012; Fang, 2016; Jin, 2013; Li et al., 2011; Li, 2019; Luo et al., 2018; Ma, 2006; Meng and Tian, 2017; Pan, 2015; Pan et al., 2013b; Pan, 2020; Peng et al., 2019; Ren et al., 2013; Schröder et al., 2022; Shen et al., 2012; Sima, 2021; Su et al., 2006; Sui et al., 2016; Wang et al., 2017; Wang et al., 2009; Wang, 2017; Wang, 2007; Wu et al., 2021; Wu et al., 2016; Xu et al., 2024; Xu et al., 2011; Yang et al., 2013; Zhang et al., 2021; Zhang, 2020; Zhao et al., 2015; Zhou et al., 2012; Zhu et al., 2017). Among these, 33 studies were published in Chinese, while two were published in English. The included studies comprised 27 journal articles, seven dissertations, and one conference paper. Sample sizes ranged from 26 to 399 participants (Table 1). The TCM treatments included in the trials were either used alone or in combination, involving 17 different Chinese herbal formulas, such as Jianpi Yifei decoction, as well as two types of Chinese herbal injections, including Shenmai injection and Jiweiling injection. Additionally, one single herb, Astragali Radix (Huangqi), was used, and seven types of non-pharmacological TCM treatments, including acupuncture, were incorporated. Conventional treatments included Riluzole, Alprazolam, Macrogol 4,000 powders, Edaravone, physical therapy, continued nursing and psychological care. Some studies reported having used integrated conventional treatment, which involves using more than two conventional treatment measures. In our study, it is referred to as “integrated conventional treatment, ICT”. Detailed information for TCM interventions is shown in Supplementary Table S1. Eleven studies only reported partial composition of the formula. Nine studies included a batch or approval number of the herb or formula, and only one study reported quality-control or standardized-production information.

**FIGURE 1:**
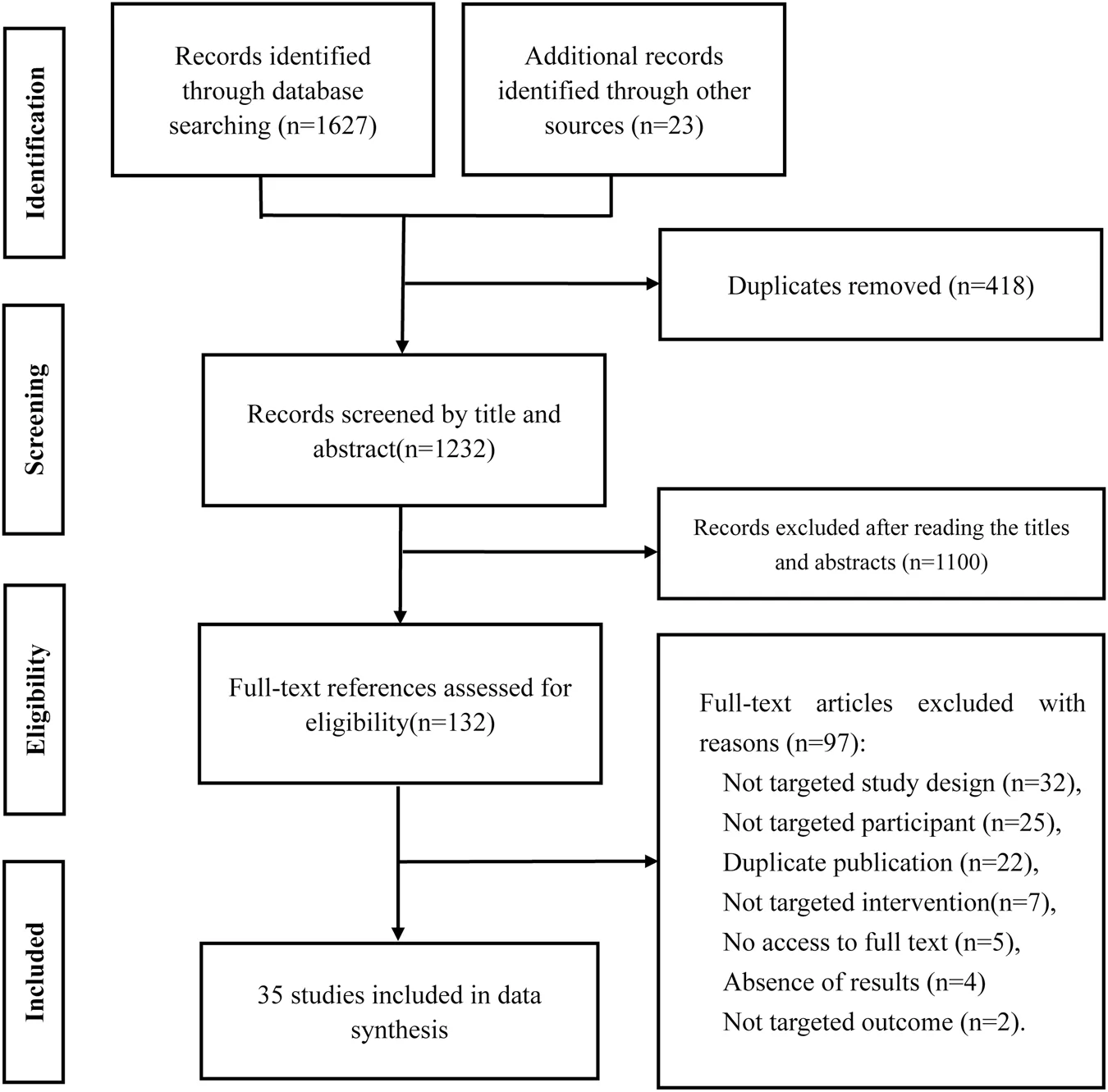
PRISMA flow diagram of study selection.

**TABLE 1:** Characteristics of included trials.

ID	Age (mean ± SD, years)		Sample size (male/female)	Course of disease (mean ± SD, months)	Treatment		Outcome measures
	T	C			T	C	
Cai et al. (2023)	56.73 ± 5.90	56.30 ± 6.58	37/23	NR	Acupuncture + rehabilitation exercises + Riluzole	Riluzole	①②
Cai RH 2011	51.2 ± 11.7	48.5 ± 9.7	20/18	T:17.1 ± 4.3 C:18.2 ± 3.3	Shenmai injection + ICT	ICT	①
Chen et al. (2004)	54.67 ± 10.23	53.56 ± 10.31	399	T: 22.44 ± 9.36 C: 22.20 ± 9.12	Jiweiling injection	ICT	⑧
Chen and Yang (2012)	44.1 ± 13.2	42.6 ± 12.7	82/38	NR	Zaolian Anshen powder + Alprazolam	Alprazolam	②
Fang ZH 2016	NR	NR	29/16	15.44 ± 9.29	Jianpi Yifei formula	Riluzole	①②③
Jin PQ 2013	57.83 ± 10.55	50.54 ± 18.85	20/8	T: 18.62 ± 11.81 C: 18.25 ± 15.81	Guilu Erxian paste + acupuncture + Riluzole	Riluzole	①②⑧
Li et al. (2011)	56.1 ± 10.8		38/20	NR	Fuyuan Shengji granules + Riluzole	Riluzole	①④⑥
Li (2019)	52.41 ± 11.48	51.08 ± 10.32	46/32	T:2.23 ± 0.78 C:2.31 ± 0.72	Jianpi Yifei formula + acupoint massage + ICT	ICT	①②
Luo et al. (2018)	49.15 ± 6.28	50.07 ± 6.31	52/24	T:2.23 ± 0.69 C:2.06 ± 0.63	Jianpi Yiqi Tongluo decoction + acupuncture + physical therapy	Acupuncture + physical therapy	③④⑧
Ma (2006)	48.33 ± 10.24	48.68 ± 11.06	19/41	T:13.32 ± 6.76 C:13.14 ± 6.52	Jiweiling injection + Riluzole placebo	Jiweiling injection placebo + Riluzole	①③④⑤⑧
Meng B 2017	52 ± 14	50 ± 16	14/14	T: 20.62 ± 12.81 C: 19.25 ± 11.91	Acupuncture + Riluzole	Riluzole	①②
Pan et al. (2013)	51.6 ± 7.2	50.1 ± 4.2	25/17	T: 25.9 ± 24.7 C: 26.1 ± 24.9	Jiawei Sijunzi decoction	Riluzole	①③⑧
Pan WR 2020	58.09 ± 9.745	56.21 ± 12.474	39/29	NR	Jiawei Sijunzi decoction + ICT	ICT	①②
Pan C 2015	49.38 ± 8.96	50.05 ± 8.10	51/25	T:13.90 ± 8.37 C:13.05 ± 8.26	Shenzhe Jiangqi powder + Riluzole placebo	Shenzhe Jiangqi powder placebo + Riluzole	①②③⑤⑧
Peng J 2019	53. 33 ± 9. 52	53. 17 ± 9. 14	19/11	T:20. 81 ± 3. 76 C:20. 14 ± 3. 36	TCM massage nursing + continued nursing	Continued nursing	①②④
Ren et al. (2013)	55.11 ± 13.52	56.64 ± 11.18	74/51	Less than 5 years	Fuyuan Shengji granules	Riluzole	①⑤⑧
Schröder S 2022	54.9 ± 7.97	53.87 ± 8.53	72/48	T:16.45 ± 5.92 C: 15.62 ± 5.09	Jiwuli plaster	Placebo	①②⑧
Shen C 2012	NR	NR	28/9	NR	Liqi Tongbian decoction + acupoint massage + acupressure	Macrogol 4,000 powders	②④⑧
Sima DD 2021	56.25 ± 13.02	56.21 ± 13.66	15/11	T:20.50 ± 12.33 C:19.86 ± 9.51	Motor neuron disease formula + ICT	ICT	①②③
Su et al. (2006)	60.2 ± 14.1	59.4 ± 9.0	19/16	NR	Yiqi Qiangji decoction + Riluzole	Riluzole	①②⑤
Sui et al. (2016)	54.00 ± 11.96	54.00 ± 11.92	38/26	NR	Huoling Shengji formula	Riluzole	①②⑧
Wang (2017)	53. 07 ± 11. 19	53. 07 ± 11. 19	32/28	Around 20 months	Jianpi Yifei formula + Riluzole	Riluzole	①②④⑧
Wang J 2009	55 ± 13 55.11 ± 13.52	55 ± 13 56.64 ± 11. 18	74/51	Less than 5 years	Fuyuan Shengji granules	Riluzole	②⑧
Wang WJ 2017	46.20 ± 6.97	48.83 ± 3.78	52/8	NR	Zishen Jianpi formula + ICT	ICT	①②③⑧
Wang XF 2007	44.57 ± 9.55	48.13 ± 8.54	42/18	T:22.20 ± 13.16 C:21.97 ± 14.59	Jiweiling injection	Riluzole	①②③④⑤⑧
Wu F 2021	53 ± 7	54 ± 8	33/27	T:2.03 ± 0.79 C:1.97 ± 0.84	Lei Huo moxibustion + Riluzole	Riluzole	①③④
Wu Y 2016	41.1	42.9	25/7	NR	Huangqi powder	Riluzole	④
Xu H 2024	60.00 ± 8.19	59.00 ± 6.53	29/15	T:12.00 (10.00,24.00) C:23.00 (12.00,24.00)	Moxibustion + Qiushi Wenshen Zhilou formula + Riluzole tablets + Edaravone injection + Routine rehabilitation nursing	Qiushi Wenshen Zhilou formula + Riluzole tablets + Edaravone injection + Routine rehabilitation nursing	①⑧
Xu et al. (2011)	NR	NR	52/28	6	Bushen Jianpi Shugan formula	Riluzole	①②
Yang et al. (2013)	46.35 ± 18.23	45.63 ± 17.52	172/68	NR	Zaolian Anshen powder + Alprazolam	Alprazolam	②⑧
Zhang et al. (2021)	47.9 ± 10.6	48.3 ± 8.9	39/23	T:27.4 ± 10.9 C:29.6 ± 19.1	Wenshen Jianpi hot compress + ICT	ICT	①②⑧
Zhang NN 2020	45.36 ± 6.52	45.51 ± 6.38	70/14	T:1.58 ± 0.32 C:1.56 ± 0.29	Shenmai injection + ICT	ICT	①
Zhao et al. (2015)	41.3 ± 10.2	42.5 ± 11.7	19/11	More than 4 months	Zaolian Anshen powder + Routine psychological care + TCM music therapy	Zaolian Anshen powder + Routine psychological care	②
Zhou et al. (2012)	76.5	71.5	23/6	NR 2–5 years	Yifei formula + acupoint massage + ICT	ICT	④⑧
Zhu et al. (2017)	NR	NR	29/16	T:3.2 ± 2.5 C:3.5 ± 2.7	Jiawei Sijunzi formula	Placebo	①②⑧

NR: not reported; ICT: integrated conventional treatment; TCM: traditional Chinese medicine; Outcomes: ①Functional status, ②Symptom improvement or relief, ③Quality of life, ④Respiratory function, ⑤Motor neuron loss, ⑥Survival, ⑦Cognitive and behavioral impairment, ⑧Adverse events/effect.

### 3.2 Risk of bias evaluation

The overall risk of bias was low in one study, raised some concerns in one study, and was high in the remaining 32 studies according to the RoB 2.0 criteria (Figure 2). All 35 included studies reported randomization, although 12 did not specify the method of sequence generation. Three studies reported explicit blinding procedures, while one study mentioned “double-blind” without providing further details. Baseline characteristics were reported as comparable between groups in all studies. Considering the overall information related to the randomization process, 31 studies were judged as having some concerns and four as low risk of bias. Four studies used placebo control with blinding, ensuring that participants and personnel were not aware of intervention assignments. However, one study randomized participants with motor neuron disease and subsequently conducted a subgroup analysis among ALS participants rather than randomizing ALS participants directly. This study was therefore judged as having a high risk of bias due to deviations from intended interventions, while the remaining studies were assessed as low risk.

**FIGURE 2:**
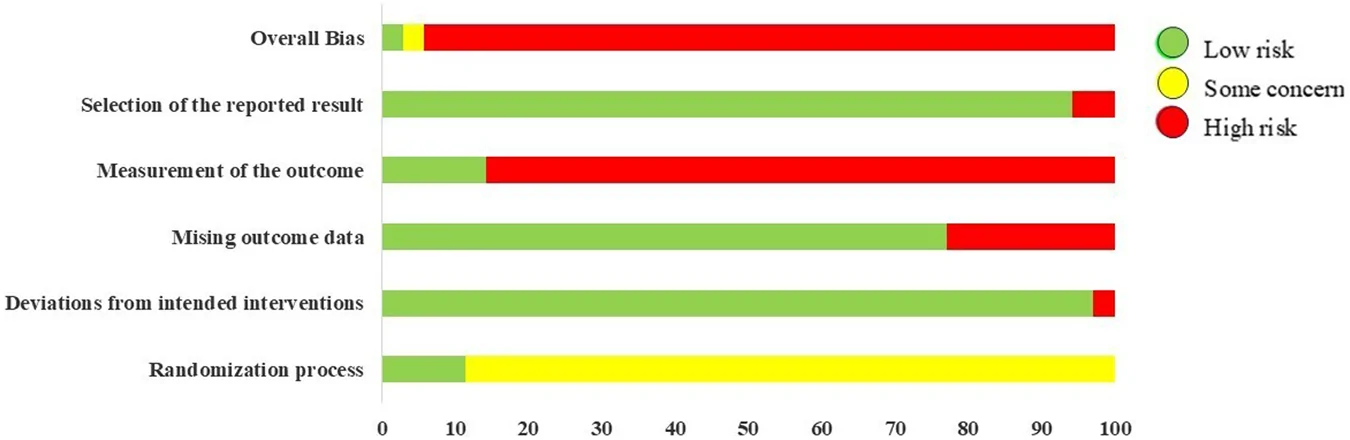
Risk of bias assessment.

Eight studies reported more than 5% loss to follow-up but did not conduct analyses to assess potential bias. In six studies, at least some cases of loss to follow-up or withdrawal were related to participants’ health status, whereas in the remaining two studies the reasons for missing data were not reported. As a result, eight studies were judged as having a high risk of bias and the remaining as low risk in missing outcome data. In measurement of the outcome, four placebo-controlled blinded studies and one additional study using objective outcomes were assessed as low risk, whereas the remaining studies were judged as high risk. In selection of the reported result, two studies showed inconsistencies between outcomes or time points reported in the methods and results sections and were therefore judged as having a high risk of bias.

### 3.3 Network meta-analysis

None of the outcome-specific networks contained a closed loop. Therefore, inconsistency between direct and indirect evidence could not be statistically tested, and a random-effects consistency model was used for all analyses.

#### 3.3.1 Functional status

##### 3.3.1.1 ALSFRS scale

As shown in Figure 3, two evidence networks were formed based on the comparisons of various treatments. One network involved four CHMs plus ICT compared to ICT, and the other compared two CHMs plus placebo with Riluzole plus placebo. The Bayesian network meta-analysis results indicated that there were no statistically significant differences between any of the treatment comparisons in terms of improving ALSFRS scores. However, the SUCRA result indicated that Shenmai injection + ICT (SUCRA score = 0.735) and Jiweiling injection + placebo (SUCRA score = 0.868) emerged as the most effective treatments within their respective networks.

**FIGURE 3:**
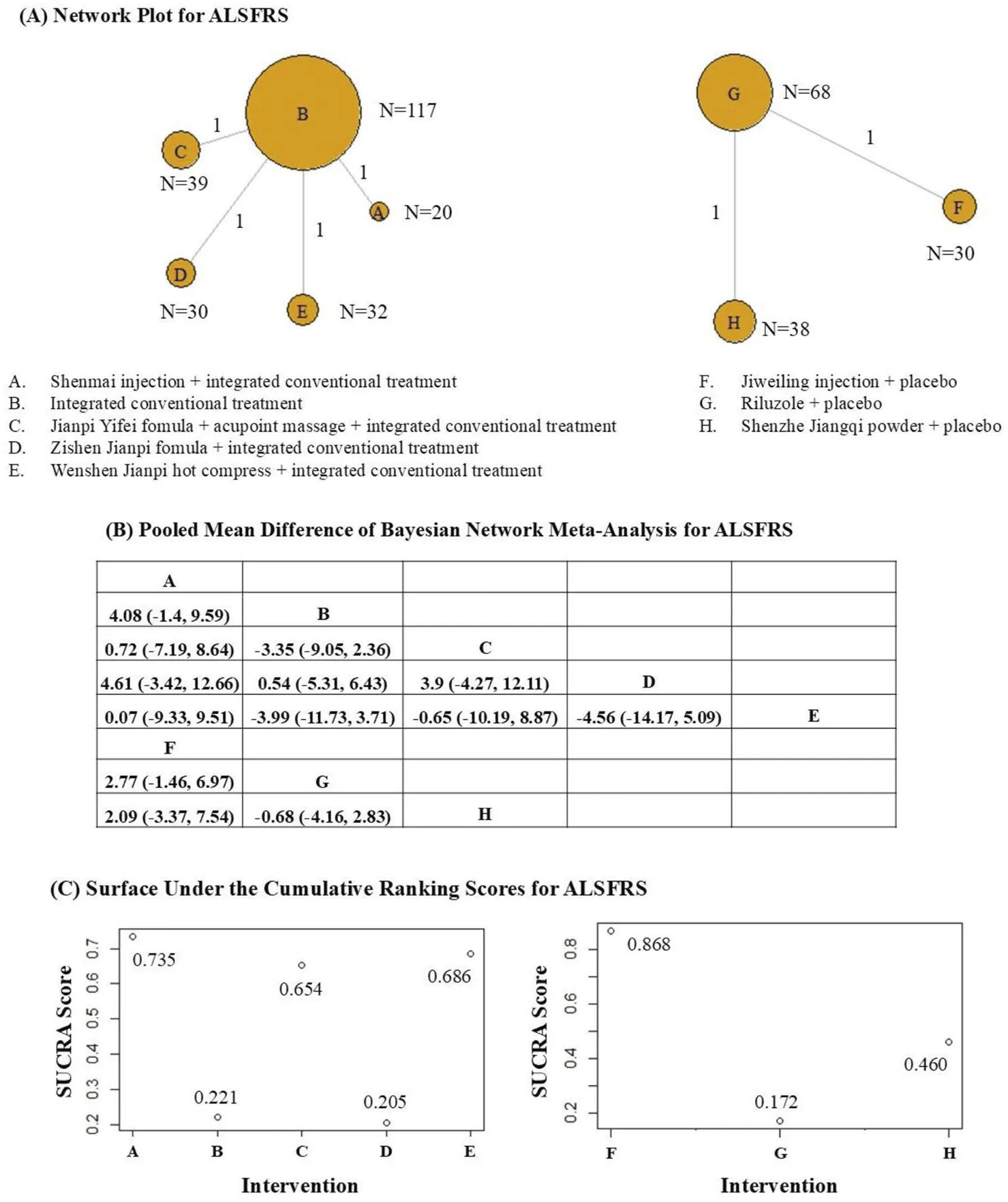
Network meta-analysis results for the ALSFRS scale. **(A)** Network Plot for ALSFRS, **(B)** Pooled Mean Difference of Bayesian Network Meta-Analysis for ALSFRS, **(C)** Surface Under the Cumulative Ranking Scores for ALSFRS.

##### 3.3.1.2 ALSFRS-R scale

Two evidence networks were constructed (Figure 4). The first network involved the comparison of three kinds of CHMs alone, two kinds of CHMs combined with Riluzole, Riluzole and placebo. The second network examined the effects of three kinds of CHMs combined with ICT compared with ICT alone. The Bayesian network meta-analysis revealed no statistically significant differences between any pair of therapies in improving ALSFRS-R scores. However, the SUCRA values indicated that Jiawei Sijunzi decoction, used as a standalone therapy, demonstrated the highest SUCRA value of 0.739. Similarly, Shenmai injection combined with ICT achieved the highest SUCRA value of 0.870.

**FIGURE 4:**
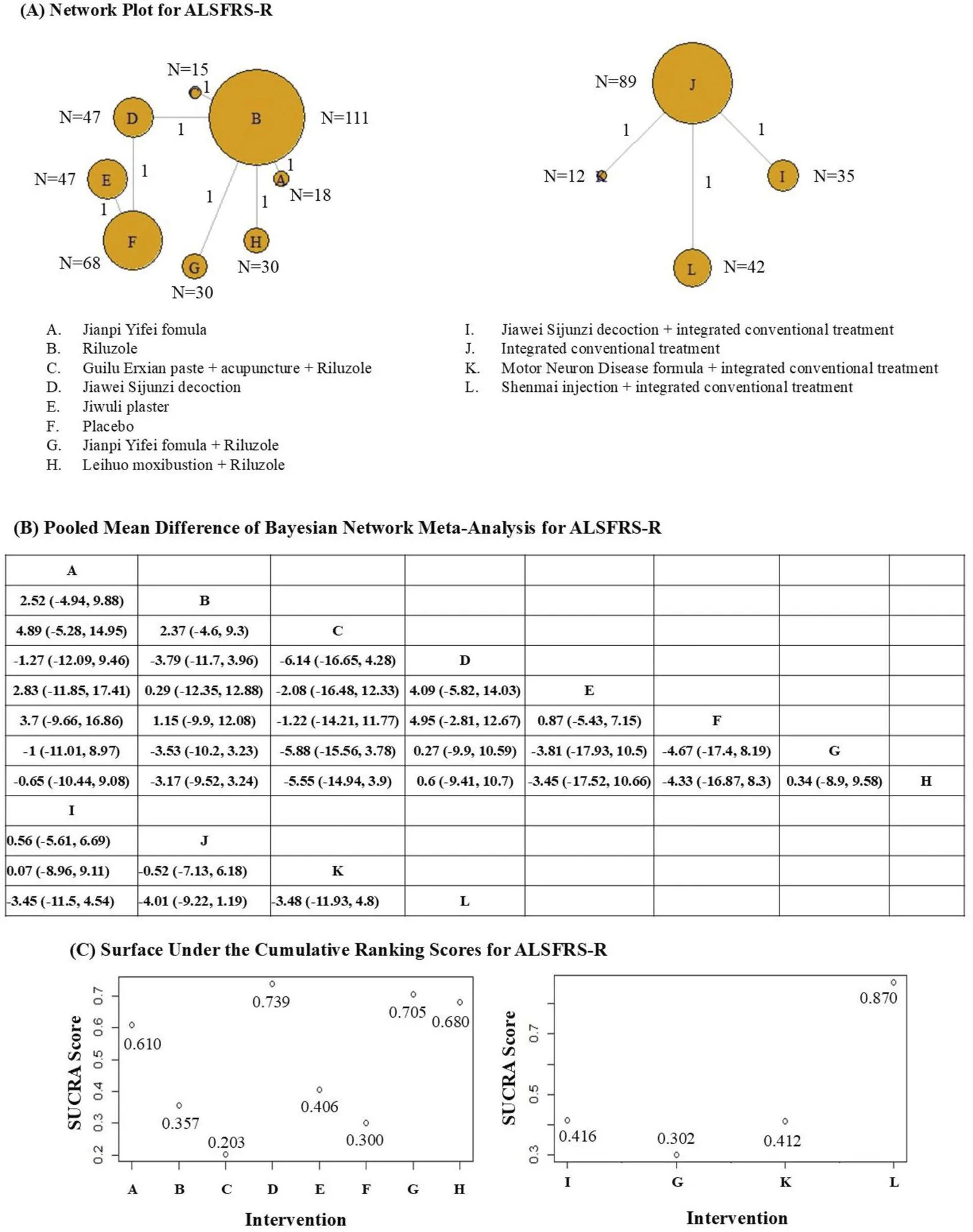
Network meta analysis results for ALSFRS-R scale. **(A)** Network Plot for ALSFRS-R, **(B)** Pooled Mean Difference of Bayesian Network Meta-Analysis for ALSFRS-R, **(C)** Surface Under the Cumulative Ranking Scores for ALSFRS-R.

##### 3.3.1.3 AALSS scale

Two studies assessed functional status using the AALSS scale (Figure 5), resulting in an evidence network comparing acupuncture + rehabilitation exercises + Riluzole, acupuncture + Riluzole, and Riluzole alone. No statistically significant differences were observed in the AALSS scores among the three treatments. The SUCRA results indicated that acupuncture + rehabilitation exercises + Riluzole was the most effective treatment option (0.928).

**FIGURE 5:**
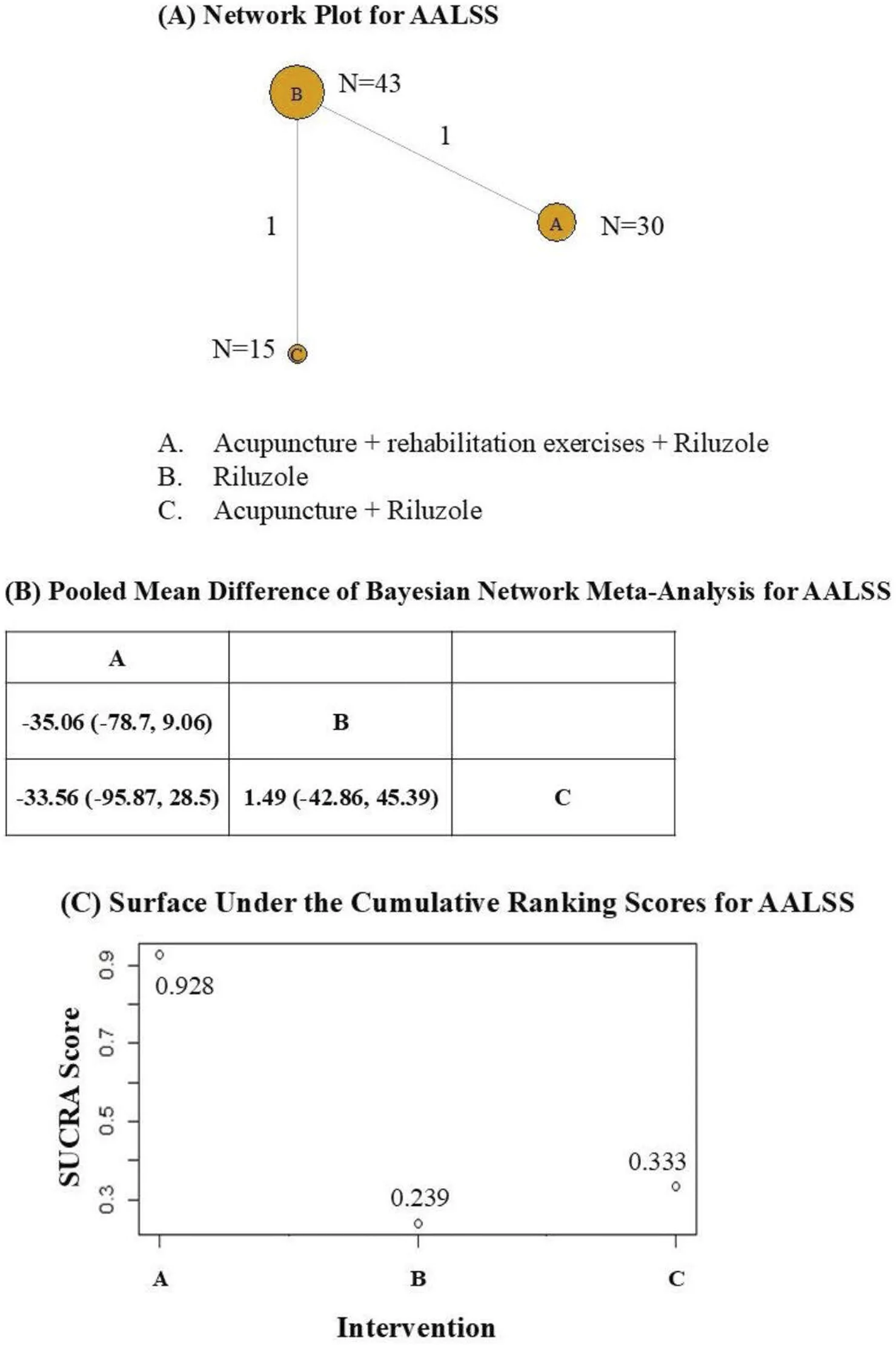
Network meta analysis results for AALSS scale. **(A)** Network Plot for AALSS, **(B)** Pooled Mean Difference of Bayesian Network Meta-Analysis for AALSS, **(C)** Surface Under the Cumulative Ranking Scores for AALSS.

##### 3.3.1.4 M-Norris Scale

A total of eight studies evaluated functional status using the m-Norris scale (Figure 6), and two network plots were constructed involving seven studies. The first network analysis compared four different TCM treatments, either alone or in combination with Riluzole, against Riluzole alone. The second network compared two TCM therapies plus placebo with Riluzole plus placebo. In both network analyses, no statistically significant differences were observed for m-Norris scale. The SUCRA results indicated that the top-ranked treatments in the two comparisons were acupuncture + rehabilitation exercises + Riluzole and Shenzhe Jiangqi powder plus placebo, with SUCRA values of 0.863 and 0.860, respectively.

**FIGURE 6:**
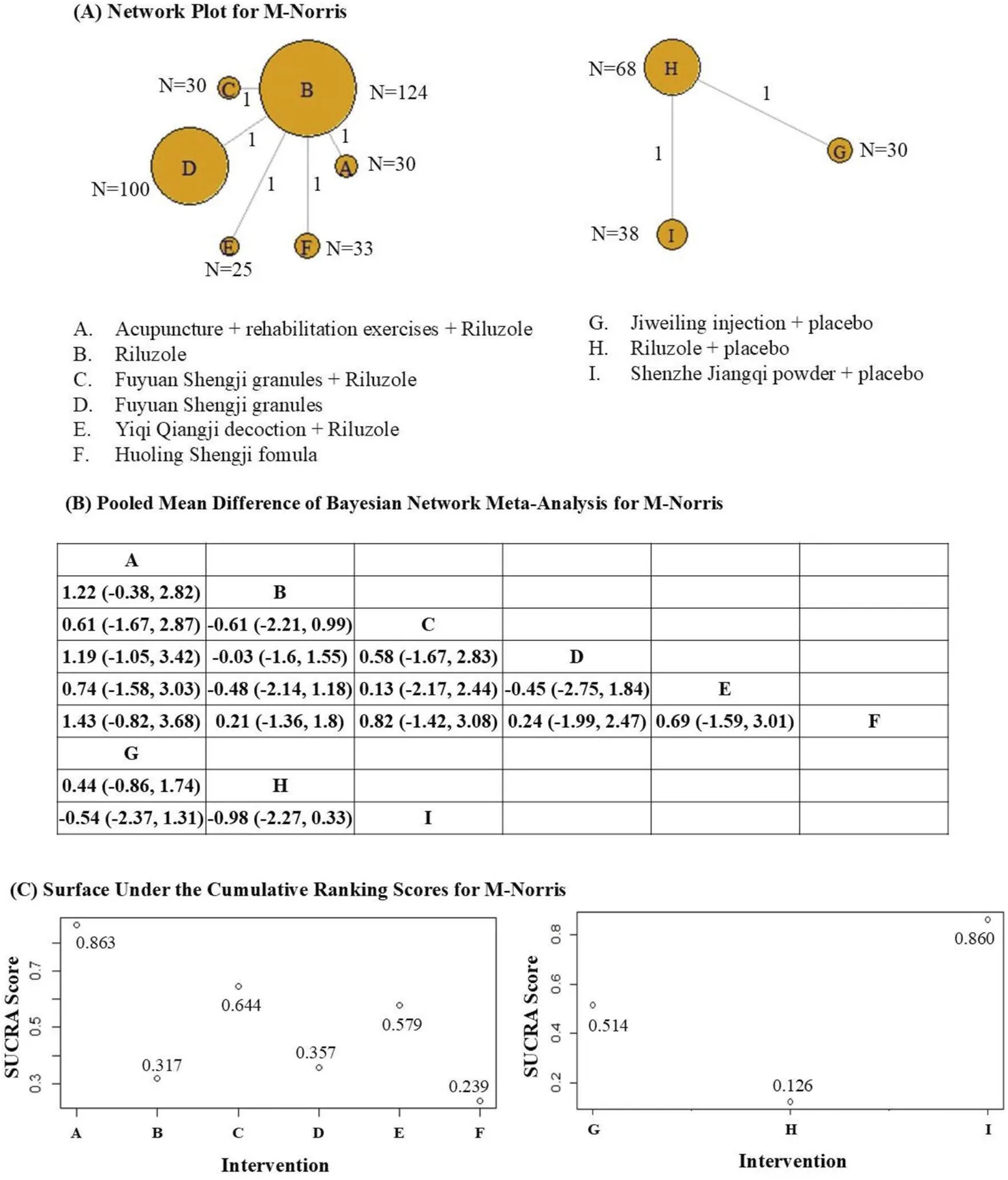
Network meta analysis results for M-Norris scale. **(A)** Network Plot for M-Norris, **(B)** Pooled Mean Difference of Bayesian Network Meta-Analysis for M-Norris, **(C)** Surface Under the Cumulative Ranking Scores for M-Norris.

#### 3.3.2 Quality of life

Five studies assessed quality of life using the ALSAQ-40 scale (Figure 7), forming two evidence networks. No statistically significant differences were observed between any pairwise comparisons of the treatments for ALSAQ-40 scale. SUCRA results indicated that Leihuo moxibustion plus Riluzole ranked first (0.846) in terms of ALSAQ-40 scale, outperforming other CHMs or Riluzole alone, while compared to Riluzole plus placebo or Shenzhe Jiangqi powder plus placebo, Jiweiling injection plus placebo ranked first (0.779).

**FIGURE 7:**
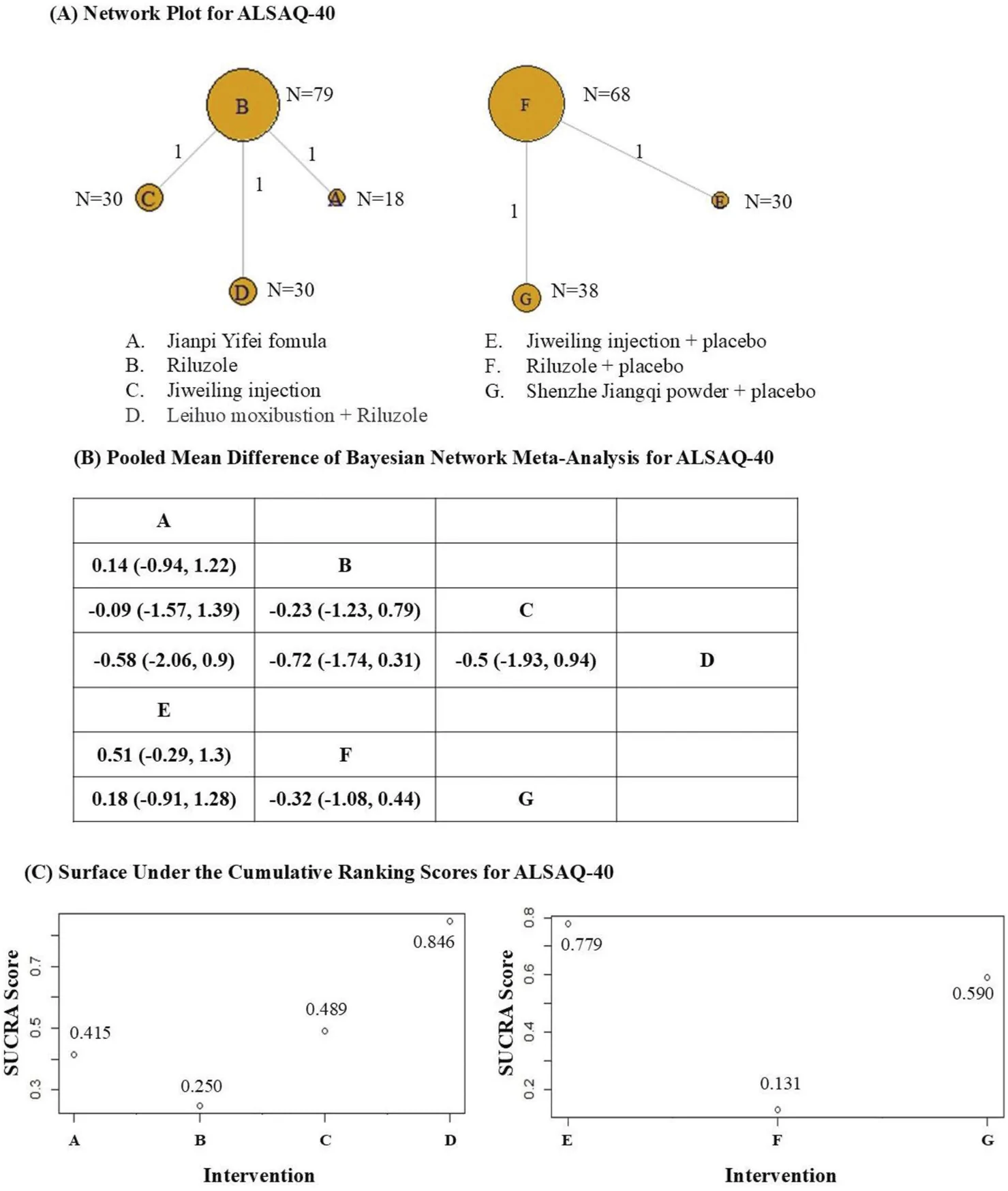
Network Meta Analysis Results for ALSAQ-40 scale. **(A)** Network Plot for ALSAQ-40, **(B)** Pooled Mean Difference of Bayesian Network Meta-Analysis for ALSAQ-40, **(C)** Surface Under the Cumulative Ranking Scores for ALSAQ-40.

### 3.4 Adverse events

Adverse events were assessed qualitatively, because pooling of adverse events of varying interventions and severity was deemed inappropriate. Our qualitative analysis revealed no apparent differences between the different intervention (Supplementary Table S2).

## 4 Discussion

### 4.1 Summary of findings

In this Bayesian network meta-analysis, we evaluated the effectiveness of specific TCM therapies for treating ALS, focusing on functional status and quality of life. A total of 35 RCTs were included, with 1,650 references initially screened. These studies involved a variety of TCM treatments, including CHMs, herbal injections, and non-pharmacological therapies like acupuncture. Our analysis revealed no statistically significant differences between TCM therapies, ICT, Riluzole alone, and Riluzole plus placebo for improving functional outcomes, as measured by ALSFRS, ALSFRS-R, AALSS, and m-Norris scales. However, the SUCRA results highlighted that certain treatments, such as Jiweiling injection, Jiawei Sijunzi decoction, Shenmai injection, Shenzhe Jiangqi powder and acupuncture plus rehabilitation exercises, demonstrated higher ranking in specific networks. Additionally, Leihuo moxibustion and Jiweiling injection had potential benefits for improving quality of life based on the ALSAQ-40 scale.

### 4.2 Agreements and disagreements with other studies

Our findings are generally consistent with previous reviews examining the effectiveness of TCM for ALS. Both Liao et al. (2022) and Song et al. (2022b) found that integrative therapies, combining TCM and conventional treatments like Riluzole, show promise in improving functional status and quality of life. Consistent with these studies, our network meta-analysis found that treatments such as Jiweiling injection, Jiawei Sijunzi decoction, Shenmai injection, Shenzhe Jiangqi powder, and acupuncture plus rehabilitation exercises ranked highest across different functional status scales, with SUCRA values indicating their superior effectiveness within specific treatment networks. The effects were consistent with pharmacological mechanisms studies. Specifically, network pharmacology analysis of the active components in Jiweiling injection identified PI3K/Akt and MAPK signaling as principal pathways regulating neuroinflammation and apoptosis (Pan et al., 2023), while Shenmai injection has been shown to protect neurons via the APJ/AMPK/GSK-3β pathway and attenuate mitochondrial dysfunction through AMPKα1 activation (Wu J. et al., 2022; Wu J. et al., 2025), with its active components ginsenoside Rb1 and Rg1 independently demonstrating inhibition of oxidative stress, apoptosis, and neuroinflammation (Gong L. et al., 2022; Zhang et al., 2022). Similarly, Astragalus polysaccharides—the principal bioactive fraction of Jiawei Sijunzi decoction—activate the PI3K/AKT/mTOR and Nrf2 pathways to stabilize mitochondrial function and reduce neuronal apoptosis (Shi and Ma, 2024), and a structurally analogous formula to Shenzhe Jiangqi powder has been shown in an ALS animal model to preserve mitochondrial integrity and improve motor function (Gong F. et al., 2022).

However, similar to these previous reviews, our study also found no statistically significant differences between treatments in terms of functional status (ALSFRS, ALSFRS-R, AALSS, m-Norris scales). This suggests that while individual TCM therapies show some effectiveness, the overall differences between treatments are minimal. Notably, our findings regarding quality of life (ALSAQ-40) outcomes did show Leihuo moxibustion + Riluzole as the most effective, consistent with the results of Song et al. (2022b). The main difference lies in our use of Bayesian network meta-analysis, which provides a more integrated approach for comparing multiple therapies simultaneously. This approach offers a more comprehensive ranking of treatments, highlighting the relative effectiveness of combined therapies over individual treatments.

### 4.3 Strengths and limitations

This study benefits from the use of Bayesian network meta-analysis, which allows for a comprehensive comparison of multiple treatments simultaneously, providing a more nuanced understanding of the relative effectiveness of different therapies for ALS. The inclusion of various treatment modalities, including TCM and conventional treatments like Riluzole, enhances the generalizability of the findings. However, there are several limitations. First, ALS is a rare disease, and the sample sizes in the included studies ranged from 26 to 399 participants, which may limit the statistical power of the analyses. The lack of statistically significant differences in functional status outcomes could be partially attributed to this limited sample size and the challenges inherent in studying rare diseases with relatively small patient populations. Importantly, SUCRA values represent the probability of a treatment being among the best options rather than statistical significance. Therefore, treatments may rank highly in SUCRA despite the absence of statistically significant differences. Additionally, the variability in treatment regimens and the diversity of TCM therapies included in the trials pose another challenge. Many of the treatments involved different combinations of TCM therapies, and no pairwise comparison in the network meta-analysis included more than two studies for any given treatment. Moreover, most comparisons in the network were informed by single studies, resulting in wide confidence intervals and limited precision, which further reduces the ability to detect statistically significant differences. This lack of consistency in treatment protocols limits the ability to draw firm conclusions about the comparative effectiveness of individual treatments. Although separate networks were constructed by outcome measures and treatment comparisons, the plausibility of transitivity remained uncertain because disease duration varied within some networks. The characterization of the TCM preparations is also insufficiently reported in the original RCTs. Many reports did not provide full botanical composition, validated taxonomic names, producer, batch number, extraction or processing parameters, or quality-control measures such as pharmacopoeial standards or chemical fingerprints. Therefore, the reproducibility and interpretability of the network estimates are limited. Finally, while we found that integrative therapies showed higher effectiveness, the heterogeneity of TCM treatments included in the analysis suggests that future research should focus on more standardized treatment protocols and well-designed trials to confirm these results and reduce variability in the treatment approaches.

### 4.4 Implications for research

The results of this study highlight the potential therapies or combinations identified through the Bayesian network meta-analysis, including TCM therapies and their combinations with conventional treatments. However, the limited sample sizes and the lack of statistically significant differences in functional outcomes underscore the challenges of conducting large-scale clinical trials in rare diseases like ALS. The small patient population makes it difficult to achieve the statistical power necessary for definitive conclusions. Given these challenges, future research should focus on building real-world databases to better understand the effectiveness and safety of these therapies in clinical settings. As noted by Liao et al. (2022) and Song et al. (2022a), real-world evidence can complement randomized controlled trials by capturing the effects of treatments across a broader, more diverse ALS population, including patients who may not meet the strict inclusion criteria for clinical trials. By integrating data from various sources, such as electronic health records and patient-reported outcomes, these databases could provide more accurate insights into the long-term benefits and risks of TCM therapies and potential treatment combinations. Additionally, future studies should focus on standardizing treatment protocols to reduce variability and enable more reliable comparisons of different therapies.

## 5 Conclusion

While no statistically significant differences were observed between treatments in terms of functional outcomes (measured by ALSFRS, ALSFRS-R, AALSS, and m-Norris scales), several treatments demonstrated higher rankings in the analysis and may warrant further investigation. Specifically, Jiweiling injection, Shenmai injection, Jiawei Sijunzi decoction and Shenzhe Jiangqi powder may be regarded as candidate preparations for future trials. Subsequent research should prioritize real-world data to further explore the long-term effects and practical benefits of these therapies.

## Data Availability

The original contributions presented in the study are included in the article/Supplementary Material, further inquiries can be directed to the corresponding author.
