# Supplementary material for: Selected traditional Chinese medicines for treating amyotrophic lateral sclerosis: a Bayesian network meta-analysis based on randomised controlled trials
Source: Front Pharmacol. 2026 Oct 8;17:1706211. doi: 10.3389/fphar.2026.1706211 (PMC13687075; doi:10.3389/fphar.2026.1706211)
Supplement: Supplementary file 1 [file Table1.docx]

**ConPhYMP assessment of the included TCM interventions**

| **Study ID** | **TCM intervention/preparation** | **Item 1: Clear title and informative abstract (Yes/No/NA)** | **Item 1 page no.** | **Item 2: Description of the botanical drug and**  **taxonomic**  **authentication (Yes/No/NA)** | **Item 2 page no.** | **Item 3: Description of the**  **extract and**  **extraction process (Yes/No/NA)** | **Item 3 page no.** | **Item 4: Documentation of the legal basis for collection and**  **processing (Yes/No/NA)** | **Item 4 page no.** | **Item 5: Commercial product batch/regulatory status (Yes/No/NA)** | **Item 5 page no.** |
| --- | --- | --- | --- | --- | --- | --- | --- | --- | --- | --- | --- |
| Cai FF 2023 | Acupuncture combined with rehabilitation exercises | NA | — | NA | — | NA | — | NA | — | NA | — |
| Cai RH 2011 | Shenmai injection | Yes | 1 | No | NR | No | NR | No | NR | No | NR |
| Chen JL 2004 | Jiweiling injection | Yes | 1 | No | NR | No | NR | No | NR | No | NR |
| Chen JL 2012 | Zaolian Anshen powder | Yes | 1 | No | NR | No | NR | No | NR | Yes | 1-2 |
| Fang ZH 2016 | Jianpi Yifei formula | Yes | 1 | No | NR | No | NR | No | NR | Yes | 21 |
| Jin PQ 2013 | Guilu Erxian paste + Acupuncture | Yes | 1 | No | NR | Yes | 1 | NA | — | NA | — |
| Li CS 2011 | Fuyuan Shengji granules | Yes | 1 | No | NR | No | NR | No | NR | No | NR |
| Li P 2019 | Jianpi Yifei formula plus acupoint massage | Yes | 1 | No | NR | Yes | 2 | NA | — | NA | — |
| Luo LN 2018 | Jianpi Yiqi Tongluo decoction plus acupuncture | Yes | 1 | No | NR | Yes | 2 | NA | — | NA | — |
| Ma WL 2006 | Jiweiling injection | Yes | 2 | No | NR | No | 13 | No | NR | Yes | 13 |
| Meng B 2017 | Acupuncture | NA | — | NA | — | NA | — | NA | — | NA | — |
| Pan Z 2015 | Shenzhe Jiangqi powder | Yes | 4 | No | NR | No | NR | NA | — | Yes | 16 |
| Pan WD 2013 | Jiawei Sijunzi decoction | Yes | 1 | No | NR | Yes | 2 | NA | — | NA | — |
| Pan WR 2020 | Jiawei Sijunzi decoction | Yes | 7 | No | NR | No | NR | NA | — | NA | — |
| Peng J 2019 | TCM massage nursing | NA | — | NA | — | NA | — | NA | — | NA | — |
| Ren HL 2013 | Fuyuan Shengji granules | Yes | 1 | No | NR | No | NR | No | NR | No | NR |
| Schröder S 2022 | Ji-Wu-Li plaster | Yes | 1 | No | NR | Yes | 4 | NA | — | Yes | Supplementary material. |
| Shen C 2012 | Liqi Tongbian decoction + acupoint massage + acupressure | Yes | 1 | No | NR | Yes | 2 | NA | — | NA | — |
| Sima DD 2021 | Motor Neuron Disease formula | Yes | 6 | No | NR | No | NR | No | NR | No | NR |
| Su GL 2006 | Yiqi Qiangji decoction | Yes | 1 | No | NR | Yes | 1 | NA | — | NA | — |
| Sui SY 2016 | Huoling Shengji formula | Yes | 1 | No | NR | Yes | 2 | NA | — | NA | — |
| Wang AQ 2017 | Jianpi Yifei formula | Yes | 1 | No | NR | No | NR | No | NR | No | NR |
| Wang J 2009 | Fuyuan Shengji granules | Yes | 1 | No | NR | No | NR | No | NR | No | NR |
| Wang WJ 2017 | Zishen Jianpi formula | Yes | 5 | No | NR | No | NR | NA | — | NA | — |
| Wang XF 2007 | Jiweiling injection | Yes | 3 | No | NR | No | NR | No | NR | Yes | 17 |
| Wu F 2021 | Lei Huo moxibustion | NA | — | NA | — | NA | — | NA | — | NA | — |
| Wu Y 2016 | Huangqi powder | Yes | 1 | No | NR | Yes | 2 | NA | — | NA | — |
| Xu H 2024 | Qiushi Wenshen Zhilou formula plus Moxibustion | Yes | 1 | No | NR | No | NR | NA | — | NA | — |
| Xu WJ 2011 | Bushen Jianpi Shugan formula | Yes | 1 | No | NR | No | NR | NA | — | NA | — |
| Yang XL 2013 | Zaolian Anshen powder | Yes | 1 | No | NR | No | NR | No | NR | Yes | 1 |
| Zhang HZ 2021 | Wenshen Jianpi hot compress | Yes | 1 | No | NR | Yes | 2 | NA | — | NA | — |
| Zhang NN 2020 | Shenmai injection | Yes | 1 | No | NR | No | NR | No | NR | Yes | 1 |
| Zhao JY 2015 | Zaolian Anshen powder with TCM music therapy | Yes | 1 | No | NR | No | NR | No | NR | Yes | 1 |
| Zhou LX 2012 | Yifei formula + Acupoint massage | Yes | 1 | No | NR | No | NR | No | NR | No | NR |
| Zhu XY 2017 | Jiawei Sijunzi formula | Yes | 1 | No | NR | No | NR | NA | — | NA | — |

NR: not reported; NA: not applicable.
