# Supplementary material for: Selected traditional Chinese medicines for treating amyotrophic lateral sclerosis: a Bayesian network meta-analysis based on randomised controlled trials
Source: Front Pharmacol. 2026 Oct 8;17:1706211. doi: 10.3389/fphar.2026.1706211 (PMC13687075; doi:10.3389/fphar.2026.1706211)
Supplement: Supplementary file 2 [file Table2.docx]

**Supplement 1. Search strategy applied in English language databases**

#1 (“amyotrophic lateral sclerosis”[MeSH Terms]) OR (“Sclerosis, Amyotrophic Lateral”[Tiab]) OR (“ALS - Amyotrophic Lateral Sclerosis”[Tiab]) OR (“ALS Amyotrophic Lateral Sclerosis”[Tiab]) OR (“Gehrig's Disease”[Tiab]) OR (“Gehrig Disease”[Tiab]) OR (“Gehrigs Disease”[Tiab]) OR (“Lou Gehrig Disease”[Tiab]) OR (“Motor Neuron Disease, Amyotrophic Lateral Sclerosis”[Tiab]) OR (“Lou Gehrig's Disease”[Tiab]) OR (“Lou-Gehrigs Disease”[Tiab]) OR (“Charcot Disease”[Tiab]) OR (“Amyotrophic Lateral Sclerosis With Dementia”[Tiab]) OR (“Dementia With Amyotrophic Lateral Sclerosis”[Tiab]) OR (“Guam Disease”[Tiab]) OR (“Disease, Guam”[Tiab]) OR (“Amyotrophic Lateral Sclerosis, Parkinsonism-Dementia Complex of Guam”[Tiab]) OR (“Amyotrophic Lateral Sclerosis, Parkinsonism Dementia Complex of Guam”[Tiab])

#2 (“medicine, traditional”[MeSH]) OR (“medicine, chinese traditional”[MeSH]) OR (“Complementary Therapies”[MeSH]) OR (“plant extracts”[MeSH]) OR (“drugs, chinese herbal”[MeSH]) OR (“Herbal Medicine”[MeSH]) OR (“Plants, Medicinal”[MeSH]) OR (“Plant preparations”[MeSH]) OR (“Medicine, Kampo”[MeSH]) OR (“Phytotherapy”[MeSH]) OR (“Acupuncture”[MeSH]) OR (“Acupuncture Therapy”[MeSH]) OR (“Chinese medicin*”[tiab]) OR (“Chinese herb*”[tiab]) OR (“Chinese plant*”[tiab]) OR (“Chinese drug*”[tiab]) OR (“Chinese formul*”[tiab]) OR (“Chinese prescri*”[tiab]) OR (“oriental medicin*”[tiab]) OR (“traditional medicin*”[tiab]) OR (“alternativ* medicin*”[tiab]) OR (“alternativ* therapy”[tiab]) OR (“complementary medicin*”[tiab]) OR (“complementary therapy”[tiab]) OR (“complementary treatment*”[tiab]) OR (“botanical extract*”[tiab]) OR (“plant extract*”[tiab]) OR (“plant preparation*”[tiab]) OR (“plant medicin*”[tiab]) OR (“herb*”[tiab]) OR (“herbal remed*”[tiab]) OR (“herbal extract*”[tiab]) OR (“herbal preparation*”[tiab]) OR (“herbal mixture*”[tiab]) OR (“herbal medicin*”[tiab]) OR (“phyto drug*”[tiab]) OR (“phyto pharmaceutical*”[tiab]) OR (“phyto therapy”[tiab]) OR (“phyto treatment*”[tiab]) OR (“phyto medicin*”[tiab]) OR (“phytopharmaceutic*”[tiab]) OR (“acupunct*”[tiab]) OR (“acupress*”[tiab]) OR (“acupoint*”[tiab]) OR (“electroacupunct*”[tiab]) OR (“auriculotherapy”[tiab]) OR (“auriculoacupunct*”[tiab]) OR (“moxibust*”[tiab]) OR (“meridian*”[tiab]) OR (“patch*”[tiab]) OR (“cupping therapy”[tiab]) OR (“Chinese massage”[tiab]) OR (“Tuina”[tiab]) OR (“scraping therapy”[tiab])

#3 #1 AND #2

**eTable 1 Information for TCM interventions reported in the included trials**

| **Study ID** | **TCM intervention** | **Intervention detail/** **composition (as reported)** |  | **Dosage/form and administration** | **Producer/manufacturer** | **Batch/approval number** | **Quality control/ pharmacopoeial standard** | **Production preparation** |
| --- | --- | --- | --- | --- | --- | --- | --- | --- |
| Cai FF 2023 | Acupuncture combined with rehabilitation exercises | - Acupoints: Jianyu (LI15), Quchi (LI11), Yangxi (LI5), Hegu (LI4), Jiaji (EX-B2), Zusanli (ST36), Jiexi (ST41), Sanyinjiao (SP6), Yanglingquan (GB34), Xuanzhong (GB39), and Weizhong (BL40). - Exercise details incomplete. |  | Acupuncture: once daily; needles retained for 10–20 min after achieving deqi sensation; 2-week treatment course. Rehabilitation exercises: performed three times daily (morning, noon, and evening). | Not applicable | Not applicable | Not applicable | Not applicable |
| Cai RH 2011 | Shenmai injection | - Panax ginseng C.A.Mey. [Araliaceae; Ginseng Radix et Rhizoma] and Ophiopogon japonicus (Thunb.) Ker Gawl. [Asparagaceae; Ophiopogonis Radix]. |  | 250 mL once daily by intravenous infusion; 15 days/course for 3 courses (3 days rest between courses). | Not reported | Not reported | Not reported | Not reported |
| Chen JL 2004 | Jiweiling injection | - Panax ginseng C.A.Mey. [Araliaceae; Ginseng Radix et Rhizoma], Cervi Cornu Pantotrichum (Lurong; animal-derived, not a botanical taxon), Cistanche deserticola Ma [Orobanchaceae; Cistanches Herba], etc. |  | 48 mL (diluted in 250 mL of 50 g/L glucose or normal saline) once daily by intravenous infusion; 25 days/course for 3 courses (5–7 days rest between courses). | Hebei Yiling Medical Pharmaceutical Factory | Not reported | Not reported | Not reported |
| Chen JL 2012 | Zaolian Anshen powder | - Ziziphus jujuba subsp. spinosa (Bunge) J.Y.Peng, X.Y.Li & L.Li [Rhamnaceae; Ziziphi Spinosae Semen], Coptis chinensis Franch. [Ranunculaceae; Coptidis Rhizoma], Bupleurum chinense DC. [Apiaceae; Bupleuri Radix], Cyperus rotundus L. [Cyperaceae; Cyperi Rhizoma], Gardenia jasminoides J.Ellis [Rubiaceae; Gardeniae Fructus], Curcuma longa L. [Zingiberaceae; Curcumae Radix], Magnetitum (Cishi; mineral, not a botanical taxon), Albizia julibrissin Durazz. [Fabaceae; Albiziae Flos], Salvia miltiorrhiza Bunge [Lamiaceae; Salviae Miltiorrhizae Radix et Rhizoma], and other herbs. |  | 5 g orally, 2-3 times daily for 4 weeks. | Hebei Yiling Hospital | Hebei Provincial Medical Institution Preparation Approval No. Z20050678 | Not reported | In-hospital preparation; process details not reported. |
| Fang ZH 2016 | Jianpi Yifei formula | - Astragalus mongholicus Bunge [Fabaceae; Astragali Radix], Codonopsis pilosula (Franch.) Nannf. [Campanulaceae; Codonopsis Radix], Atractylodes macrocephala Koidz. [Asteraceae; Atractylodis Macrocephalae Rhizoma], Poria (Fuling), Ophiopogon japonicus (Thunb.) Ker Gawl. [Asparagaceae; Ophiopogonis Radix], Schisandra chinensis (Turcz.) Baill. [Schisandraceae; Schisandrae Chinensis Fructus], Citrus reticulata Blanco [Rutaceae; Citri Reticulatae Pericarpium], Pinellia ternata (Thunb.) Makino [Araceae; Pinelliae Rhizoma Praeparatum], Iris domestica (L.) Goldblatt & Mabb. [Iridaceae; Belamcandae Rhizoma], Acorus tatarinowii Schott [Acoraceae; Acori Tatarinowii Rhizoma], Bombyx Batryticatus (Jiangcan; animal-derived, not a botanical taxon), Sauromatum giganteum (Engl.) Cusimano & Hett. [Araceae; Typhonii Rhizoma], Strychnos nux-vomica L. [Loganiaceae; Strychni Semen], Glycyrrhiza uralensis Fisch. [Fabaceae; Glycyrrhizae Radix et Rhizoma Praeparata cum Melle], etc. |  | Granule, 1 sachet daily, dissolved in 250 mL of warm water and administered orally for 12-week treatment course. | Jiangyin Tianjiang Pharmaceutical Co., Ltd. | Batch No. 1401105 | Not reported | Not reported |
| Jin PQ 2013 | Guilu Erxian paste + Acupuncture | - Guilu Erxian paste: Cervi Cornu Degelatinatum (Lujiaoshuang; animal-derived, not a botanical taxon) 12 g, Testudinis Carapax et Plastrum (Guijia; animal-derived, not a botanical taxon) 20 g, Panax ginseng C.A.Mey. [Araliaceae; Ginseng Radix et Rhizoma] 15 g, Lycium barbarum L. [Solanaceae; Lycii Fructus] 20 g. - Acupoints: Fengchi (GB20), Weishu (BL21), Pishu (BL20), Ganshu (BL18), Shenshu (BL23), Jiaji (EX-B2), Jiansanzhen, Quchi (LI11), Hegu (LI4), Biguan (ST31), Futu (ST32), Zusanli (ST36), Sanyinjiao (SP6), and Taichong (LR3). |  | Formula: 1 dose daily, decocted twice and taken morning/evening; nasogastric administration if dysphagia.  Acupuncture: Once daily, with needles retained for 30 minutes each session; the even reinforcing-reducing manipulation (ping bu ping xie) was applied. | Not applicable | Not applicable | Not reported | Self-prepared herbal decoction; decocted twice for 1 dose |
| Li CS 2011 | Fuyuan Shengji granules | - Astragalus mongholicus Bunge [Fabaceae; Astragali Radix], Atractylodes macrocephala Koidz. [Asteraceae; Atractylodis Macrocephalae Rhizoma], Cornus officinalis Siebold & Zucc. [Cornaceae; Corni Fructus], and other herbs. |  | 1 sachet administered orally twice daily for a 6-month treatment course. | Not reported | Not reported | Not reported | Not reported |
| Li P 2019 | Jianpi Yifei formula plus acupoint massage | - Formula: Astragalus mongholicus Bunge [Fabaceae; Astragali Radix] 30–60 g, Codonopsis pilosula (Franch.) Nannf. [Campanulaceae; Codonopsis Radix] 15–30 g, Atractylodes macrocephala Koidz. [Asteraceae; Atractylodis Macrocephalae Rhizoma] 15 g, Eucommia ulmoides Oliv. [Eucommiaceae; Eucommiae Cortex], 12 g, Cuscuta chinensis Lam. [Convolvulaceae; Cuscutae Semen] 12 g, Ophiopogon japonicus (Thunb.) Ker Gawl. [Asparagaceae; Ophiopogonis Radix] 9 g, Pinellia ternata (Thunb.) Makino [Araceae; Pinelliae Rhizoma Praeparatum] 9 g, Aster tataricus L.f. [Asteraceae; Asteris Radix et Rhizoma] 9 g, Prunus armeniaca L. [Rosaceae; Armeniacae Semen Amarum] 9 g, Platycodon grandiflorus (Jacq.) A.DC. [Campanulaceae; Platycodonis Radix] 9 g, Zingiber officinale Roscoe [Zingiberaceae; Zingiberis Rhizoma] 6 g, Schisandra chinensis (Turcz.) Baill. [Schisandraceae; Schisandrae Chinensis Fructus] 6 g, Citrus reticulata Blanco [Rutaceae; Citri Reticulatae Pericarpium] 6 g, Bombyx Batryticatus (Jiangcan; animal-derived, not a botanical taxon) 6 g, Bupleurum chinense DC. [Apiaceae; Bupleuri Radix] 6 g, Glycyrrhiza uralensis Fisch. [Fabaceae; Glycyrrhizae Radix et Rhizoma Praeparata cum Melle] 6 g and Strychnos nux-vomica L. [Loganiaceae; Strychni Semen] 0.1 g. - Acupoints: Gaohuangshu (BL43), Pishu (BL20), Xinshu (BL15), Shenzhu (GV12), Feishu (BL13), and Dingchuan (EX-B1) |  | Formula: 1 dose daily, taken warm (re-decocted and administered a second time the same day; given in divided doses or via nasogastric tube for patients with dysphagia), for 3 months.  Acupoint massage: once daily (2 minutes per acupoint), for 3 months. | Not applicable | Not applicable | Not reported | Self-prepared herbal decoction; decocted in water over low heat to 200 mL. |
| Luo LN 2018 | Jianpi Yiqi Tongluo decoction plus acupuncture | - Formula: Astragalus mongholicus Bunge [Fabaceae; Astragali Radix] 25 g, Atractylodes macrocephala Koidz. [Asteraceae; Atractylodis Macrocephalae Rhizoma], Angelica sinensis (Oliv.) Diels [Apiaceae; Angelicae Sinensis Radix], Bupleurum chinense DC. [Apiaceae; Bupleuri Radix] and Citrus reticulata Blanco [Rutaceae; Citri Reticulatae Pericarpium] 10 g each; Panax ginseng C.A.Mey. [Araliaceae; Ginseng Radix et Rhizoma], Carthamus tinctorius L. [Asteraceae; Carthami Flos], Glycyrrhiza uralensis Fisch. [Fabaceae; Glycyrrhizae Radix et Rhizoma Praeparata cum Melle], Paeonia lactiflora Pall. [Paeoniaceae; Paeoniae Radix Alba] and Atractylodes lancea (Thunb.) DC. [Asteraceae; Atractylodis Rhizoma] 6 g each, Alisma plantago-aquatica subsp. orientale (Sam.) Sam. [Alismataceae; Alismatis Rhizoma] 15 g. - Acupoints: Shousanli (LI10), Zusanli (ST36), Biguan (ST31), Jiexi (ST41), Futu (ST32), Quchi (LI11), Jianyu (LI15), Hegu (LI4), Dazhui (GV14), Yinlingquan (SP9), Pishu (BL20), Ganshu (BL18), Shenshu (BL23), Shenzhu (GV12), and Jiaji (EX-B2). |  | Formula: 1 dose daily, 150 mL taken orally each morning and evening (300 mL/day), for a 3-month treatment course.  Acupuncture: once daily; 8–12 acupoints selected according to disease condition. Needles were retained for 0.5 h. Reinforcing manipulation was applied at Sanyinjiao (SP6) and Taixi (KI3); balanced reinforcing-reducing manipulation at Fengfu (GV16), Fengchi (GB20), and Lianquan (CV23); reducing manipulation at Neiting (ST44) and Fenglong (ST40). Warm needle moxibustion was applied to the remaining acupoints (2 moxa cones per acupoint) for 0.5 h. | Not applicable | Not applicable | Not reported | Decocted in water to 300 mL. Self-prepared decoction. |
| Ma WL 2006 | Jiweiling injection | - Panax ginseng C.A.Mey. [Araliaceae; Ginseng Radix et Rhizoma], Eucommia ulmoides Oliv. [Eucommiaceae; Eucommiae Cortex], Cuscuta chinensis Lam. [Convolvulaceae; Cuscutae Semen], Salvia miltiorrhiza Bunge [Lamiaceae; Salviae Miltiorrhizae Radix et Rhizoma], Angelica sinensis (Oliv.) Diels [Apiaceae; Angelicae Sinensis Radix], and Cinnamomum aromaticum Nees [Lauraceae; Cinnamomi Cortex]. |  | 32 mL diluted in 250 mL of 5% glucose or 0.9% saline, once daily by intravenous infusion; 28 days/course. | Preparation Unit of Hebei Yiling Hospital. | Approval number: Shi-Wei-Yao-Ji-Zi (98) Mie 501-00296 | Not reported | In-hospital preparation; prepared by hospital preparation unit (process details not reported). |
| Meng B 2017 | Acupuncture | - Shenshu (BL23), Yaoshu (GV2), Yaoyangguan (GV3), Mingmen (GV4), Jizhong (GV6), Zhongshu (GV7), Jinsuo (DU8), Zhiyang (GV9), Lingtai (GV10), Shendao (GV11), Shenzhu (GV12), Taodao (GV13), Dazhui (GV14), Baihui (GV20), Shenting (GV24). |  | Needle retention 30 min per session; once daily; 2 months/course for 3 courses. | Not applicable | Not applicable | Not applicable | Not applicable |
| Pan C 2015 | Shenzhe Jiangqi powder | - Codonopsis pilosula (Franch.) Nannf. [Campanulaceae; Codonopsis Radix], Haematitum (Zheshi; mineral, not a botanical taxon), Testudinis Carapax et Plastrum (Guiban; animal-derived, not a botanical taxon); Cistanche deserticola Ma [Orobanchaceae; Cistanches Herba], Cornus officinalis Siebold & Zucc. [Cornaceae; Corni Fructus], Dioscorea polystachya Turcz. [Dioscoreaceae; Dioscoreae Rhizoma]; Platycodon grandiflorus (Jacq.) A.DC. [Campanulaceae; Platycodonis Radix], Citrus aurantium L. [Rutaceae; Aurantii Fructus]; Impatiens balsamina L. [Balsaminaceae; Impatientis Semen]; Scolopendra (Wugong; animal-derived, not a botanical taxon). |  | 5 g orally per dose, three times daily; 90 days. | Hebei Yiling Hospital. | Approval number: Ji-Yao-Zhi-Zi Z20050674 | Not reported | In-hospital preparation; specific processing steps not reported. |
| Pan WD 2013 | Jiawei Sijunzi decoction | - Panax ginseng C.A.Mey. [Araliaceae; Ginseng Radix et Rhizoma] 9 g, Astragalus mongholicus Bunge [Fabaceae; Astragali Radix] 30 g, Cistanche deserticola Ma [Orobanchaceae; Cistanches Herba] 12 g, Atractylodes macrocephala Koidz. [Asteraceae; Atractylodis Macrocephalae Rhizoma] 9 g, Poria (Fuling) 9 g, Glycyrrhiza uralensis Fisch. [Fabaceae; Glycyrrhizae Radix et Rhizoma] 9 g. |  | Decoction 50 mL administered orally twice daily for 6 months. | Manufacturing laboratory of Shuguang Hospital. | Not applicable | Not reported | All herbs were placed into 400 mL cold water, soaked for 30 min, then boiled over low flame for 30 min to obtain approximately 100 mL decoction; hospital laboratory preparation. |
| Pan WR 2020 | Jiawei Sijunzi decoction | - Codonopsis pilosula (Franch.) Nannf. [Campanulaceae; Codonopsis Radix] 20 g, Astragalus mongholicus Bunge [Fabaceae; Astragali Radix Praeparata cum Melle] 20 g, Poria (Fuling) 10 g, Atractylodes macrocephala Koidz. [Asteraceae; Atractylodis Macrocephalae Rhizoma] 10 g, Glycyrrhiza uralensis Fisch. [Fabaceae; Glycyrrhizae Radix et Rhizoma Praeparata cum Melle] 10 g, Angelica sinensis (Oliv.) Diels [Apiaceae; Angelicae Sinensis Radix] 10 g. |  | 1 dose daily, administered orally, 3 months/course for 3 courses, total 9 months. | Not reported | Not reported | Not reported | Detailed preparation method not reported. |
| Peng J 2019 | TCM massage nursing | - Manipulations along the Jiaji region and Du meridian, abdominal and limb manipulations, and acupoint stimulation including Jiquan (HT1), Zusanli (ST36), Yanglingquan (GB34), Kunlun (BL60), Taixi (KI3), Jiexi (ST41), Chongyang (ST42), and Taichong (LR3), with ankle dorsiflexion and Achilles tendon stretching. |  | Once daily; 5 consecutive days per week with 2 days rest; intervention duration 3 months. | Not applicable | Not applicable | Not reported | No medicinal product preparation involved. |
| Ren HL 2013 | Fuyuan Shengji granules | - Not reported. |  | 15 g per dose, orally, twice daily, for 12 weeks. | Self-prepared in-hospital Chinese patent medicine (exact manufacturer name not reported). | Not reported | Not reported | In-hospital granule preparation; detailed manufacturing process not reported. |
| Schröder S 2022 | Ji-Wu-Li plaster | - Panax ginseng C.A.Mey. [Araliaceae; Ginseng Radix et Rhizoma]; Astragalus mongholicus Bunge [Fabaceae; Astragali Radix], Cistanche deserticola Ma [Orobanchaceae; Cistanches Herba], Atractylodes macrocephala Koidz. [Asteraceae; Atractylodis Macrocephalae Rhizoma], Poria (Fuling), Glycyrrhiza uralensis Fisch. [Fabaceae; Glycyrrhizae Radix et Rhizoma], Rhodiola crenulata (Hook.f. & Thomson) H.Ohba [Crassulaceae; Rhodiolae Crenulatae Radix et Rhizoma], Epimedium sagittatum (Siebold & Zucc.) Maxim. [Berberidaceae; Epimedii Folium]. Total herbal content 32 g/plaster; ratio 2:6:3:2:2:2:2:2. |  | 90 × 135 mm plaster placed on the midline back below the spinous process of the seventh cervical vertebra (C7); retained 6 h/day for 6 consecutive days followed by 1 day rest; repeated for 20 weeks. | Shanghai NUANYOU Industry Co. Ltd. | Batch number: Ginseng Radix 20210305; Astragali Radix 210804; Cistanchis Radix Herba 210202; Atractylodis Macrocephalae Rhizoma 210622; Poria 210710; Glycyrrhizae Radix 210624; Rhodiola Rosea Radix 210524. Approval number: Not reported. | Identity and purity were verified per Chinese Pharmacopoeia 2020 (Part I / enterprise internal standards) or Shanghai TCM Decoction Pieces Processing Specification (2018), depending on herb. Independent residue and organic trace analyses (e.g., C202103-002-002; FL-03-002-00) confirmed heavy metals, pesticide residues, and microbiological contamination were within pharmacopoeial limits. | Purified raw herbs were crushed and passed through an 80-mesh sieve, mixed with a hot-melt adhesive matrix, and then assembled into a plaster with a heating layer containing iron powder, salt, and activated carbon. |
| Shen C 2012 | Liqi Tongbian decoction + acupoint massage + acupressure | - Decoction: Pleuropterus multiflorus (Thunb.) Turcz. ex Nakai [Polygonaceae; Polygoni Multiflori Radix] 20 g, Citrus aurantium L. [Rutaceae; Aurantii Immaturus] 12 g, Raphanus raphanistrum subsp. sativus (L.) Schmalh. [Brassicaceae; Raphani Semen] 15 g, Rheum officinale Baill. [Polygonaceae; Rhei Radix et Rhizoma] 9 g, Areca catechu L. [Arecaceae; Arecae Semen] 12 g, Atractylodes macrocephala Koidz. [Asteraceae; Atractylodis Macrocephalae Rhizoma] 10 g; Astragalus mongholicus Bunge [Fabaceae; Astragali Radix], Ageratum conyzoides L. [Asteraceae, [agerati conyzoidii herbae](https://mpns.science.kew.org/mpns-portal/drugDetail?drugName=agerati+conyzoidii+herbae&query=huoxiang&filter=&fuzzy=false&nameType=all)] 6 g. - Acupoint massage: One-finger Zen pushing and rubbing manipulation was applied to the lower abdomen, focusing on Shenque (CV8), Tianshu (ST25), Zhongwan (CV12), and Qihai (CV6). A suitable amount of talcum powder was applied to the left lower abdomen, followed by repeated pushing massage along the ascending colon, transverse colon, descending colon, and sigmoid colon, with gradually increased amplitude to produce an abdominal depression of about 1 cm. Manipulation was slow, gentle, and deep. - Acupressure: Acupoints included Tianshu (ST25) and Zhigou (TE6). Both thumbs were used to apply gradually increasing pressure from light to heavy until soreness/distension, tenderness, and bowel peristalsis were elicited. Each press was maintained for several seconds up to 1 min; alternating repeated pressure was applied when a single press was insufficient. |  | Decoctions: 1 dose/day, decocted in water; administered via nasogastric tube in 2–3 divided doses/day, 100 mL each time, for 14 days.  Acupoint massage: Once daily, 10 min per session, performed 30 min after breakfast or 20 min before defecation.  Acupressure: 3-5 min per acupoint; performed daily, typically before defecation (about 10 min/session). | Not reported | Not reported | Not reported | Water decoction prepared for enteral administration. |
| Sima DD 2021 | Motor Neuron Disease formula | - Cuscuta chinensis Lam. [Convolvulaceae; Cuscutae Semen] 15 g, Epimedium sagittatum (Siebold & Zucc.) Maxim. [Berberidaceae; Epimedii Folium] 15 g, Cistanche deserticola Ma [Orobanchaceae; Cistanches Herba] 30 g, Rehmannia glutinosa (Gaertn.) Libosch. ex DC. [Orobanchaceae; Rehmanniae Radix] 30 g, Cornus officinalis Siebold & Zucc. [Cornaceae; Corni Fructus] 25 g, Crocus sativus L. [Iridaceae; Croci Stigma] 1 g, Astragalus mongholicus Bunge [Fabaceae; Astragali Radix] 60 g, Panax ginseng C.A.Mey. [Araliaceae; Ginseng Radix et Rhizoma] 6 g, Bupleurum chinense DC. [Apiaceae; Bupleuri Radix] 9 g, Actaea cimicifuga L. [Ranunculaceae; Cimicifugae Rhizoma] 9 g. |  | 90-day treatment; exact dosage form/administration not fully reported. | Not reported | Not reported | Not reported | Detailed preparation not reported. |
| Su GL 2006 | Yiqi Qiangji decoction | - Astragalus mongholicus Bunge [Fabaceae; Astragali Radix] 60 g, Codonopsis pilosula (Franch.) Nannf. [Campanulaceae; Codonopsis Radix] 30 g, Epimedium sagittatum (Siebold & Zucc.) Maxim. [Berberidaceae; Epimedii Folium] 30 g, Poria (Fuling) 20 g, Rehmannia glutinosa (Gaertn.) Libosch. ex DC. [Orobanchaceae; Rehmanniae Radix] 20 g, Rheum officinale Baill. [Polygonaceae; Rhei Radix et Rhizoma Praeparata] 6 g, Actaea cimicifuga L. [Ranunculaceae; Cimicifugae Rhizoma] 20 g, Glycyrrhiza uralensis Fisch. [Fabaceae; Glycyrrhizae Radix et Rhizoma] 10 g. |  | 1 dose daily, water decoction, orally administered twice daily, morning/evening, for 3 months. | Not reported | Not reported | Not reported | Daily decoction from raw herbs; 1 dose/day, divided oral administration. |
| Sui SY 2016 | Huoling Shengji formula | - Epimedium sagittatum (Siebold & Zucc.) Maxim. [Berberidaceae; Epimedii Folium], Astragalus mongholicus Bunge [Fabaceae; Astragali Radix], Atractylodes macrocephala Koidz. [Asteraceae; Atractylodis Macrocephalae Rhizoma], Cornus officinalis Siebold & Zucc. [Cornaceae; Corni Fructus], Poria (Fuling), Rehmannia glutinosa (Gaertn.) Libosch. ex DC. [Orobanchaceae; Rehmanniae Radix]. Individual herb doses not reported. |  | Oral decoction; 200 mL/day divided into 100 mL twice daily; 12 weeks. | Not reported | Not reported | Not reported | Daily decoction from raw herbs; 1 dose/day, divided oral administration. |
| Wang AQ 2017 | Jianpi Yifei formula plus riluzole | - Astragalus mongholicus Bunge [Fabaceae; Astragali Radix] 30 g; Codonopsis pilosula (Franch.) Nannf. [Campanulaceae; Codonopsis Radix] 15g, Atractylodes macrocephala Koidz. [Asteraceae; Atractylodis Macrocephalae Rhizoma] 15 g, Schisandra chinensis (Turcz.) Baill. [Schisandraceae; Schisandrae Chinensis Fructus] 10 g, Eucommia ulmoides Oliv. [Eucommiaceae; Eucommiae Cortex] 10 g, Cuscuta chinensis Lam. [Convolvulaceae; Cuscutae Semen] 10 g, Ophiopogon japonicus (Thunb.) Ker Gawl. [Asparagaceae; Ophiopogonis Radix] 10 g, Citrus reticulata Blanco [Rutaceae; Citri Reticulatae Pericarpium] 10 g, Pinellia ternata (Thunb.) Makino [Araceae; Pinelliae Rhizoma Praeparatum] 10 g, Bombyx Batryticatus (Jiangcan; animal-derived, not a botanical taxon) 10 g, Prunus armeniaca L. [Rosaceae; Armeniacae Semen Amarum] 10 g, Platycodon grandiflorus (Jacq.) A.DC. [Campanulaceae; Platycodonis Radix] 5 g; Bupleurum chinense DC. [Apiaceae; Bupleuri Radix] 5 g, Strychnos nux-vomica L. [Loganiaceae; Strychni Semen] 0.2 g, Glycyrrhiza uralensis Fisch. [Fabaceae; Glycyrrhizae Radix et Rhizoma Praeparata cum Melle] 10 g. |  | Oral granules; 1 dose/day dissolved in water (may be split into 2 doses if needed); 8 weeks. | Not reported | Not reported | Not reported | Not reported |
| Wang J 2009 | Fuyuan Shengji granules | - Hospital-made granule; herb composition not reported. |  | Oral granules; 15 g twice daily; 12 weeks. | Shuguang Hospital Affiliated to Shanghai University of TCM | Not reported | Not reported | In-house hospital granule preparation; no detailed process reported. |
| Wang WJ 2017 | Zishen Jianpi formula | - Rehmannia glutinosa (Gaertn.) Libosch. ex DC. [Orobanchaceae; Rehmanniae Radix Praeparata] 30 g, Gynochthodes officinalis (F.C.How) Razafim. & B.Bremer [Rubiaceae; Morindae Officinalis Radix] 15 g, Dendrobium officinale Kimura & Migo [Orchidaceae; Dendrobii Caulis] 20 g; Cistanche deserticola Ma [Orobanchaceae; Cistanches Herba] 30 g, Schisandra chinensis (Turcz.) Baill. [Schisandraceae; Schisandrae Chinensis Fructus] 20 g, Ophiopogon japonicus (Thunb.) Ker Gawl. [Asparagaceae; Ophiopogonis Radix] 20 g, Astragalus mongholicus Bunge [Fabaceae; Astragali Radix] 30 g, Atractylodes macrocephala Koidz. [Asteraceae; Atractylodis Macrocephalae Rhizoma] 30 g, Citrus reticulata Blanco [Rutaceae; Citri Reticulatae Pericarpium] 15 g, Bupleurum chinense DC. [Apiaceae; Bupleuri Radix] 10 g, Acorus tatarinowii Schott [Acoraceae; Acori Tatarinowii Rhizoma] 12 g, Polygala tenuifolia Willd. [Polygalaceae; Polygalae Radix] 10 g, Mentha canadensis L. [Lamiaceae; Menthae Haplocalycis Herba] 10 g, Platycodon grandiflorus (Jacq.) A.DC. [Campanulaceae; Platycodonis Radix] 20 g, Zingiber officinale Roscoe [Zingiberaceae; Zingiberis Rhizoma] 3 slices. Individualized additions reported. |  | Oral decoction; 1 dose/day divided morning and evening; 1 month/course. | Not reported | Not reported | Not reported | Not reported |
| Wang XF 2007 | Jiweiling injection | - Injection product; crude-drug composition not reported. Product specification: 1 mL contains 0.9 g crude drug equivalent; 8 mL/ampoule. |  | 24 mL added to 250 mL 5% glucose or 0.9% sodium chloride; once daily by intravenous infusion; 28 days/course. | Hebei Yiling Pharmaceutical Co., Ltd. | Approval number: Shi Wei Yao Ji Zi (98) 501-00296; batch number not reported. | Not reported | Commercial injection; 8 mL ampoules.. |
| Wu F 2021 | Lei Huo moxibustion | - Acupoints: Feishu (BL13), Gaohuang (BL43), Pishu (BL20), Weishu (BL21), bilateral. Moxibustion stick: Zhao’s thunder-fire moxibustion stick 2.8 cm × 10 cm. |  | Suspended moxibustion; 10 min/point; every other day; 3 months. | Zhao's Lei Hu Moxibustion Traditional Medicine Research Institute. | Not reported | Not reported | Commercial Lei Huo moxibustion stick. |
| Wu Y 2016 | Huangqi powder | - Astragalus mongholicus Bunge [Fabaceae; Astragali Radix] only. |  | 60 g/day, taken orally with warm water in three divided doses; 3 months. | Not reported | Not reported | Not reported | Oral crude powder |
| Xu H 2024 | Qiushi Wenshen Zhilou formula plus Moxibustion | - Decoction: Codonopsis pilosula (Franch.) Nannf. [Campanulaceae; Codonopsis Radix] 30 g, Atractylodes macrocephala Koidz. [Asteraceae; Atractylodis Macrocephalae Rhizoma] 15 g, Dioscorea polystachya Turcz. [Dioscoreaceae; Dioscoreae Rhizoma] 15 g, Rehmannia glutinosa (Gaertn.) Libosch. ex DC. [Orobanchaceae; Rehmanniae Radix Praeparata] 15 g, Cornus officinalis Siebold & Zucc. [Cornaceae; Corni Fructus] 12 g, Epimedium sagittatum (Siebold & Zucc.) Maxim. [Berberidaceae; Epimedii Folium] 12 g, Cervi Cornus Colla (Lujiaojiao; animal-derived, not a botanical taxon) 9 g, Cinnamomum aromaticum Nees [Lauraceae; Cinnamomi Cortex] 6 g, Alpinia oxyphylla Miq. [Zingiberaceae; Alpiniae Oxyphyllae Fructus] 10 g, Scorpio (Quanxie; animal-derived, not a botanical taxon) 3 g.   Moxibustion: Governor Vessel moxibustion from Changqiang (GV1) to Dazhui (GV14); concurrent moxa-box moxibustion at Quchi (LI11) and Zusanli (ST36). Du-moxibustion powder: Cinnamomum aromaticum Nees [Lauraceae; Cinnamomi Cortex], Conioselinum anthriscoides 'Chuanxiong' [Apiaceae; Chuanxiong Rhizoma], Asarum heterotropoides F.Schmidt [Aristolochiaceae; Asari Radix et Rhizoma], and Gaultheria crenulata Kurz [Ericaceae; Spatholobi Caulis] in equal parts. |  | Oral decoction; 1 dose/day divided into 150 mL twice daily; 3 months.  Moxibustion once weekly, 2 weeks/course for 3 months. | Decoction uniformly prepared by the Hospital pharmacy of the First Affiliated Hospital of Zhejiang Chinese Medical University;  Du-moxibustion powder: not specified; mulberry-bark paper: Hangzhou Leiou Medical Device Co., Ltd. | Batch for herbs/decoction not reported.  Mulberry-bark paper batch no. 22030707-1; | Not reported | Hospital pharmacy–prepared decoction. |
| Xu WJ 2011 | Bushen Jianpi Shugan formula | - Astragalus mongholicus Bunge [Fabaceae; Astragali Radix], Rehmannia glutinosa (Gaertn.) Libosch. ex DC. [Orobanchaceae; Rehmanniae Radix Praeparata], Epimedium sagittatum (Siebold & Zucc.) Maxim. [Berberidaceae; Epimedii Folium], Gynochthodes officinalis (F.C.How) Razafim. & B.Bremer [Rubiaceae; Morindae Officinalis Radix], Cornus officinalis Siebold & Zucc. [Cornaceae; Corni Fructus], Poria (Fuling), Dendrobium officinale Kimura & Migo [Orchidaceae; Dendrobii Caulis], Achyranthes bidentata Blume [Amaranthaceae; Achyranthis Bidentatae Radix], Bupleurum chinense DC. [Apiaceae; Bupleuri Radix], Curcuma longa L. [Zingiberaceae; Curcumae Radix]， etc. |  | Oral decoction; 1 dose/day divided into 2 doses; 6 months. | Not reported | Not reported | Not reported | Empirical hospital decoction. |
| Yang XL 2013 | Zaolian Anshen powder | - Ziziphus jujuba subsp. spinosa (Bunge) J.Y.Peng, X.Y.Li & L.Li [Rhamnaceae; Ziziphi Spinosae Semen]; Coptis chinensis Franch. [Ranunculaceae; Coptidis Rhizoma], Bupleurum chinense DC. [Apiaceae; Bupleuri Radix], Cyperus rotundus L. [Cyperaceae; Cyperi Rhizoma], Gardenia jasminoides J.Ellis [Rubiaceae; Gardeniae Fructus], Curcuma longa L. [Zingiberaceae; Curcumae Radix], Magnetitum (Cishi; mineral, not a botanical taxon), Albizia julibrissin Durazz. [Fabaceae; Albiziae Flos], Salvia miltiorrhiza Bunge [Lamiaceae; Salviae Miltiorrhizae Radix et Rhizoma], etc. |  | Oral powder; 5 g per dose, 2–3 times/day; 4 weeks. | Hebei Yiling Hospital (in-house TCM preparation) | Approval number: Ji Yao Zhi Zi Z20050678; batch number not reported. | Not reported | Hospital-made proprietary powder |
| Zhang HZ 2021 | Wenshen Jianpi hot compress | - Panax ginseng C.A.Mey. [Araliaceae; Ginseng Radix et Rhizoma] 10 parts, Astragalus mongholicus Bunge [Fabaceae; Astragali Radix] 30 parts, Cistanche deserticola Ma [Orobanchaceae; Cistanches Herba] 15 parts, Atractylodes macrocephala Koidz. [Asteraceae; Atractylodis Macrocephalae Rhizoma] 10 parts, Poria (Fuling) 10 parts, Glycyrrhiza uralensis Fisch. [Fabaceae; Glycyrrhizae Radix et Rhizoma] 10 parts, Rhodiola crenulata (Hook.f. & Thomson) H.Ohba [Crassulaceae; Rhodiolae Crenulatae Radix et Rhizoma] 10 parts, Epimedium sagittatum (Siebold & Zucc.) Maxim. [Berberidaceae; Epimedii Folium] 10 parts. |  | Transdermal hot patch 90 mm × 135 mm; 1 patch/day below Dazhui (DU14) for 6 h, then horizontally around Shenshu (BL23) area for 2–4 h; 6 days on / 1 day off; 4 weeks/course × 4 courses (~4 months). | Powder prepared by Shuguang Hospital pharmacy; patch manufactured by Shanghai Nuanyou Industrial Co., Ltd. | Not reported | Not reported | Powder extracted using standardized production technology, transdermal agent added, made into plaster; heating granules added. |
| Zhang NN 2020 | Shenmai injection | - Contains Panax ginseng C.A.Mey. [Araliaceae; Ginseng Radix et Rhizoma] and Ophiopogon japonicus (Thunb.) Ker Gawl. [Asparagaceae; Ophiopogonis Radix], etc. |  | 20 mL Shenmai Injection added to 250 mL 5% glucose; once daily intravenous infusion; 15 days/course × 3 courses with 3-day interval between courses. | Zhengda Qingchunbao Pharmaceutical Co., Ltd. | Approval number: Z33020022; batch number not reported. | Not reported | Commercial injection; 5 mL/vial. |
| Zhao JY 2015 | Zaolian Anshen powder with TCM music therapy | - Formula: Ziziphus jujuba subsp. spinosa (Bunge) J.Y.Peng, X.Y.Li & L.Li [Rhamnaceae; Ziziphi Spinosae Semen]; Coptis chinensis Franch. [Ranunculaceae; Coptidis Rhizoma], Bupleurum chinense DC. [Apiaceae; Bupleuri Radix], Cyperus rotundus L. [Cyperaceae; Cyperi Rhizoma], Gardenia jasminoides J.Ellis [Rubiaceae; Gardeniae Fructus], Curcuma longa L. [Zingiberaceae; Curcumae Radix], Magnetitum (Cishi; mineral, not a botanical taxon), Albizia julibrissin Durazz. [Fabaceae; Albiziae Flos], Salvia miltiorrhiza Bunge [Lamiaceae; Salviae Miltiorrhizae Radix et Rhizoma], etc. - Music therapy: patient-selected music 20–30 min twice daily (40–50 dB). |  | Oral powder 5 g three times daily; 4 weeks.  Music therapy: patient-selected music, 20–30 min twice daily | Hebei Yiling Hospital (in-house TCM preparation) | Approval number: Ji Yao Zhi Zi Z20050678 | Not reported | Hospital-made proprietary powder. |
| Zhou LX 2012 | Yifei formula + Acupoint massage | - Formula: Sedi Herba (Chuipencao) 30 g, Glycyrrhiza uralensis Fisch. [Fabaceae; Glycyrrhizae Radix et Rhizoma] 6 g, Citrus aurantium L. [Rutaceae; Aurantii Fructus] 10 g, Asini Corii Colla (Ejiao) 10 g, Coix lacryma-jobi L. [Poaceae; Coicis Semen] 30 g, Atractylodes macrocephala Koidz. [Asteraceae; Atractylodis Macrocephalae Rhizoma] 10 g, Poria (Fuling) 30 g, Scutellaria baicalensis Georgi [Lamiaceae; Scutellariae Radix] 10 g, Curcuma longa L. [Zingiberaceae; Curcumae Radix] 10 g, Bupleurum chinense DC. [Apiaceae; Bupleuri Radix] 6 g; Salvia miltiorrhiza Bunge [Lamiaceae; Salviae Miltiorrhizae Radix et Rhizoma] 20 g Paeonia lactiflora Pall. [Paeoniaceae; Paeoniae Radix Alba] 10 g, Panax ginseng C.A.Mey. [Araliaceae; Ginseng Radix et Rhizoma] 3 g. - Acupoint massage: Shenzhu (GV12); Feishu (BL13); Xinshu (BL15); Pishu (BL20); Gaohuang (BL43); Dingchuan (EX-B1), etc.; massage from Tianzhu (BL10) to Shenshu (BL23) region. |  | Yifei formula Nasogastric decoction; 100 mL per administration, 2–3 times/day; 14 days.  Acupoint massage: Once daily before meals; ~2 min/point; 14 days. | Zhejiang Provincial Hospital of TCM & Western Medicine (in-house formula) | Not reported | Not reported | In-house decoction |
| Zhu XY 2017 | Jiawei Sijunzi formula | - Codonopsis pilosula (Franch.) Nannf. [Campanulaceae; Codonopsis Radix] 20g, Astragalus mongholicus Bunge [Fabaceae; Astragali Radix] 20 g, Atractylodes macrocephala Koidz. [Asteraceae; Atractylodis Macrocephalae Rhizoma] 10 g, Poria (Fuling) 10 g, Glycyrrhiza uralensis Fisch. [Fabaceae; Glycyrrhizae Radix et Rhizoma Praeparata cum Melle] 10 g, Angelica sinensis (Oliv.) Diels [Apiaceae; Angelicae Sinensis Radix] 10 g. |  | 1 dose/day dissolved in water; 9 months. | Not reported | Not reported | Not reported | Not reported |

**eTable 2. Adverse events of the included studies.**

| **Study** | **Intervention** | **Control** | **Complications of the intervention group** | **Complications of the control group** |
| --- | --- | --- | --- | --- |
| Cai FF 2023 | Acupuncture + Rehabilitation exercises + Riluzole | Riluzole | No adverse events/effects. | No adverse events/effects. |
| Chen JL 2004 | Jiweiling injection | ICT | No adverse events/effects. | No adverse events/effects. |
| Jin PQ 2013 | Guilu Erxian paste + acupuncture + Riluzole | Riluzole | No adverse events/effects. | No adverse events/effects. |
| Luo LN 2018 | Jianpi Yiqi Tongluo decoction + acupuncture + physical therapy | Acupuncture + physical therapy | No adverse events/effects. | No adverse events/effects. |
| Ma WL 2006 | Jiweiling injection + Riluzole placebo | Jiweiling injection placebo + Riluzole | No adverse events/effects. | Abnormal liver function, mainly characterized by increased transaminase levels (n=22) 73.33% |
| Pan WD 2013 | Jiawei Sijunzi decoction | Riluzole | Nausea (n=2) 8.70%  Constipation (n=2) 8.70% | Nausea (n=9) 47.37%  Dizziness (n=6) 31.58%  Anorexia (n=7) 36.84%  Constipation (n=3) 15.79%  Diarrhea (n=3) 15.79%  Drug related liver/kidney damage (n=4) 21.05% |
| Pan C 2015 | Shenzhu Jiangqi powder + Riluzole placebo | Shenzhu Jiangqi Powder placebo + Riluzole | No adverse events/effects. | Elevated transaminase levels (n=13) 34.21%  Nausea (n=5) 13.16%  Fatigue (n=11) 28.95% |
| Ren HL 2013 | Fuyuan Shengji granules | Riluzole | No adverse events/effects. | NR |
| Schröder S 2022 | Jiwuli plaster | Placebo | Local allergic dermatitis (n=10) 16.67%  Fever (n=12) 20%  Sore throat (n=8) 13.33%  Nausea (n=11) 18.33%  Constipation (n=9) 15% | Local allergic dermatitis (n=9) 15%  Fever (n=11) 18.33%  Sore throat (n=9) 15%  Nausea (n=9) 15%  Constipation (n=7) 11.67% |
| Shen C 2012 | Liqi Tongbian decoction + acupoint massage + Acupressure | Macrogol 4000 powders | Diarrhea (n=2) 8.70% | No adverse events/effects. |
| Sui SY 2016 | Huoling Shengji formula | Riluzole | Nausea (n=1) 3.03%  Bloating (n=2) 6.06% | Nausea (n=8) 25.81%  Decreased appetite (n=4) 12.90%  Bloating (n=9) 29.03%  Diarrhea (n=2) 6.45% |
| Wang AQ 2017 | Jianpi Yifei formula + Riluzole | Riluzole | Local allergic reactions (n=3) 10.00%  Gastrointestinal disorders (n=1) 3.33%  Liver function abnormalities (n=2) 6.66% | Local allergic reactions (n=4)  Gastrointestinal disorders (n=1) 3.33%  Liver function abnormalities (n=1) 3.33% |
| Wang J 2009 | Fuyuan Shengji granules | Riluzole | No adverse events/effects. | NR |
| Wang WJ 2017 | Zishen Jianpi formula + ICT | ICT | No adverse events/effects. | NR |
| Wang XF 2007 | Jiweiling injection | Riluzole | No adverse events/effects. | Abnormal liver function mainly characterized by increased transaminase levels (n=13) 43.33% |
| Xu H 2024 | Moxibustion + Qiushi Wenshen Zhilou formula + ICT | Qiushi Wenshen Zhilou formula + ICT | Constipation (n=3) 13.64%  Dry mouth and sore throat (n=1) 4.55%  Skin itching (n=2) 9.09%  Skin rash (n=1) 4.55% | Constipation (n=2) 9.09%  Dry mouth and sore throat (n=2) 9.09%  Skin itching (n=1) 4.55%  Skin rash (n=2) 9.09% |
| Yang XL 2013 | Zaolian Anshen powder + Alprazolam | Alprazolam | No adverse events/effects. | No adverse events/effects. |
| Zhang HZ 2021 | Wenshen Jianpi hot compress + ICT | ICT | Itching of the skin | No adverse events/effects. |
| Zhou LX 2012 | Yifei formula + acupoint massage + ICT | ICT | No significant adverse effects | No significant adverse effects |
| Zhu XY 2016 | Jiawei Sijunzi formula | Placebo | No significant adverse effects | No significant adverse effects |

NR: not reported; ICT: Integrated conventional treatment
